# High‐Throughput Single‐Cell Analysis of Local Nascent Protein Deposition in 3D Microenvironments via Extracellular Protein Identification Cytometry (EPIC)

**DOI:** 10.1002/adma.202415981

**Published:** 2024-12-04

**Authors:** Marieke Meteling, Castro Johnbosco, Alexis Wolfel, Francisco Conceição, Kannan Govindaraj, Liliana Moreira Teixeira, Jeroen Leijten

**Affiliations:** ^1^ Leijten Laboratory Department of Developmental Bioengineering Faculty of Science and Technology, Technical Medical Centre University of Twente Drienerlolaan 5 Enschede 7522NB The Netherlands; ^2^ Department of Advanced Organ bioengineering and Therapeutics Faculty of Science and Technology, Technical Medical Centre University of Twente Drienerlolaan 5 Enschede 7522NB The Netherlands

**Keywords:** cell–cell communication, engineered microniche, extracellular matrix, high‐throughput analysis, microfluidics, micromaterials, single‐cell analysis

## Abstract

Extracellular matrix (ECM) guides cell behavior and tissue fate. Cell populations are notoriously heterogeneous leading to large variations in cell behavior at the single‐cell level. Although insights into population heterogeneity are valuable for fundamental biology, regenerative medicine, and drug testing, current ECM analysis techniques only provide either averaged population‐level data or single‐cell data from a limited number of cells. Here, extracellular protein identification cytometry (EPIC) is presented as a novel platform technology that enables high‐throughput measurements of local nascent protein deposition at single‐cell level. Specifically, human primary chondrocytes are microfluidically encapsulated in enzymatically crosslinked microgels of 16 picoliter at kHz rates, forming large libraries of discrete 3D single‐cell microniches in which ECM can be deposited. ECM proteins are labeled using fluorescence immunostaining to allow for nondestructive analysis via flow cytometry. This approach reveals population heterogeneity in matrix deposition at unprecedented throughput, allowing for the identification and fluorescent activated cell sorting‐mediated isolation of cellular subpopulations. Additionally, it is demonstrated that inclusion of a second cell into microgels allows for studying the effect of cell‐cell contact on matrix deposition. In summary, EPIC enables high‐throughput single‐cell analysis of nascent proteins in 3D microenvironments, which is anticipated to advance fundamental knowledge and tissue engineering applications.

## Graphical abstract

Knowledge of the extracellular matrix drives the understanding of cell behavior. However, current analysis methods are limited to either bulk or low‐throughput single‐cell analysis, thus masking the heterogeneity in matrix deposition. Extracellular protein identification cytometry (EPIC) combines the high‐throughput single‐cell analysis of flow cytometry with engineered microniche 3D cell culture, jointly enabling in situ matrix analysis of large cell populations.

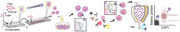

## 1 Introduction

Extracellular matrix (ECM) is imperative for complex life. It plays an essential role in the formation and maintenance of tissues, and instructs cell behavior. In particular, a cell engages in reciprocal processes to construct and modify its microenvironment. It deposits and breaks down ECM components, while simultaneously interacting with its microenvironment (including the ECM) through a complex network of signaling pathways, which collectively influence the cell's internal programming, and determine cell behavior, including the production of ECM proteins.^[^ ^1^ ^]^ Changes in the ECM thus cause changes in cell behavior and vice versa, which can lead to alternations on the tissue level. As such, nascent ECM deposition is often used as a gold standard biomarker for the development of engineered living tissues. Aberrant deposition or remodeling of ECM in tissues, such as local matrix stiffening, or excessive degradation of matrix proteins, is associated with various diseases, such as osteoarthritis,^[^ ^2^ ^]^ cancer cell growth,^[^ ^3^ ^]^ and fibrosis.^[^ ^4^ ^]^ To fully understand the fundamentals of these diseases, developmental processes, and tissue regeneration, an analysis and integration of the deposited ECM and the respective cell's microenvironment information is necessary.^[^ ^5^ ^]^ Although it is widely accepted that virtually all cell populations are naturally heterogeneous, the extend and (regenerative/pathogenic) role of heterogeneity in cell populations regarding their deposition and remodeling of nascent matrix proteins has unfortunately remained poorly understood.

Historically, conventional methods for analyzing ECM deposition have been limited to bulk measurements that obscure population heterogeneity. Consequently, several highly relevant single‐cell proteomics approaches have recently been developed including advanced fluorescence microscopy,^[^ ^6^ ^]^ single‐cell mass spectrometry,^[^ ^7^ ^]^ metabolic labeling of nascent ECM,^[^ ^1b^ ^]^ microengraving,^[^ ^8^ ^]^ single‐cell western blots,^[^ ^9^ ^]^ and spatial proteomics,^[^ ^10^ ^]^ which have particularly advanced our ability to gain insights into spatial protein variation within tissues. However, despite these significant advances, they associate with strict limitations in terms of compatibility with single‐cell nascent protein analysis. Specifically, they are incompatible with deposited matrix protein analysis (e.g., limited to cytoplasmic proteins, cell markers, and cytokines^[^ ^11^ ^]^), inherently destructive (e.g., mass spectrometry, western blot, and mass cytometry^[^ ^11^, ^12^ ^]^), limited to a specific class of matrix proteins such as collagen's (e.g., Raman spectroscopy or second harmonic imaging^[^ ^6^ ^]^), or are limited in their power to identify (rare) subpopulations due to low sampling power. Consequently, a method to nondestructively study the heterogeneity of ECM deposition at single‐cell resolution for substantial numbers of cells has remained elusive, hampering our ability to enable the identification and isolation of (living) cellular subpopulations based on variations in ECM deposition.

Although flow cytometry is the most commonly used technique to measure a plethora of different biomarkers at single‐cell resolution in high‐throughput, it is currently incompatible with measuring deposited ECM due to the required enzymatic digestion and/or physical removal of ECM to detach adherent cells for measurements. We hypothesized that this challenge could be resolved by encapsulating individual cells within micrometer‐small engineered microniches (e.g., microgels). Specifically, this allows for mass 3D suspension cell culture of adherent cells, with each individual cell in a separate physical environment that complies with matrix deposition. For ECM detection, the engineered microniches, including deposited/remodeled ECM, can be passed intact through a flow cytometer thus circumventing the traditional necessity for matrix destruction.

Single‐cell encapsulation can be achieved by employing microfluidic devices such as flow focus droplet generators. Although several such platforms have been developed,^[^ ^13^ ^]^ the explored applications have been limited to short‐term studies as they relied on emulsions composed of cell containing aqueous droplets within a persistent oil phase,^[^ ^13a,e^ ^]^ microgels that associate with rapid cell egression,^[^ ^13b,f^ ^]^ or microgels of relatively large sizes (e.g., >100 µm diameter),^[^ ^14^ ^]^ each of which challenges flow cytometry analysis of deposited matrix. Fortunately, recent developments have made it possible to microfluidically coat individual cells with a conformal micrometer‐thin layer of hydrogel in a long‐term stable (i.e., little to no cell egression over month‐long time periods) and cytocompatible manner.^[^ ^13b^ ^]^ We hypothesized that this technical innovation could enable the long‐awaited use of flow cytometry to measure deposited nascent ECM proteins.

Here, we introduce extracellular protein identification cytometry (EPIC), as a novel platform technology for high‐throughput measurements of matrix protein deposition and matrix remodeling in engineered 3D microniches at the single‐cell level. This was accomplished by combining microfluidically generated small (<40 µm) cell‐laden microgels with flow cytometry and using fluorescence immunostaining to label ECM proteins. Cell retention over time was ensured by the use of an in‐house developed cell‐centering microfluidic encapsulation approach, which minimizes cell egression over time,^[^ ^13b^ ^]^ and thus allows for the long‐term cell culture necessary to realize nascent ECM deposition. Furthermore, the cell encapsulation created a micrometer‐thin 3D microenvironment around the cell, which could be tuned to contain one or more cells, hence enabling the study of the influence of cell–cell contact on nascent ECM deposition. The small size of the microgels uniquely enabled fast diffusion of immunolabels allowing for efficient protocols, while offering high signal to noise ratios. Moreover, the small microgel size enabled in situ analysis of immunolabeled deposited ECM in high‐throughput at single‐cell resolution via its inherent compatibility with flow cytometry. In summary, we present a novel approach that delivers single‐cell analysis of deposited ECM in high‐throughput in a nondestructive manner. We anticipate that EPIC will aid in the development and deeper analysis of engineered living tissues, cell‐based therapies, personalized medicine, and drug screening models.

## 2 Results and Discussion

### 2.1 Engineering Single‐Cell 3D Microniches for Stable Long‐Term Single‐Cell Culture

**Figure 1:**
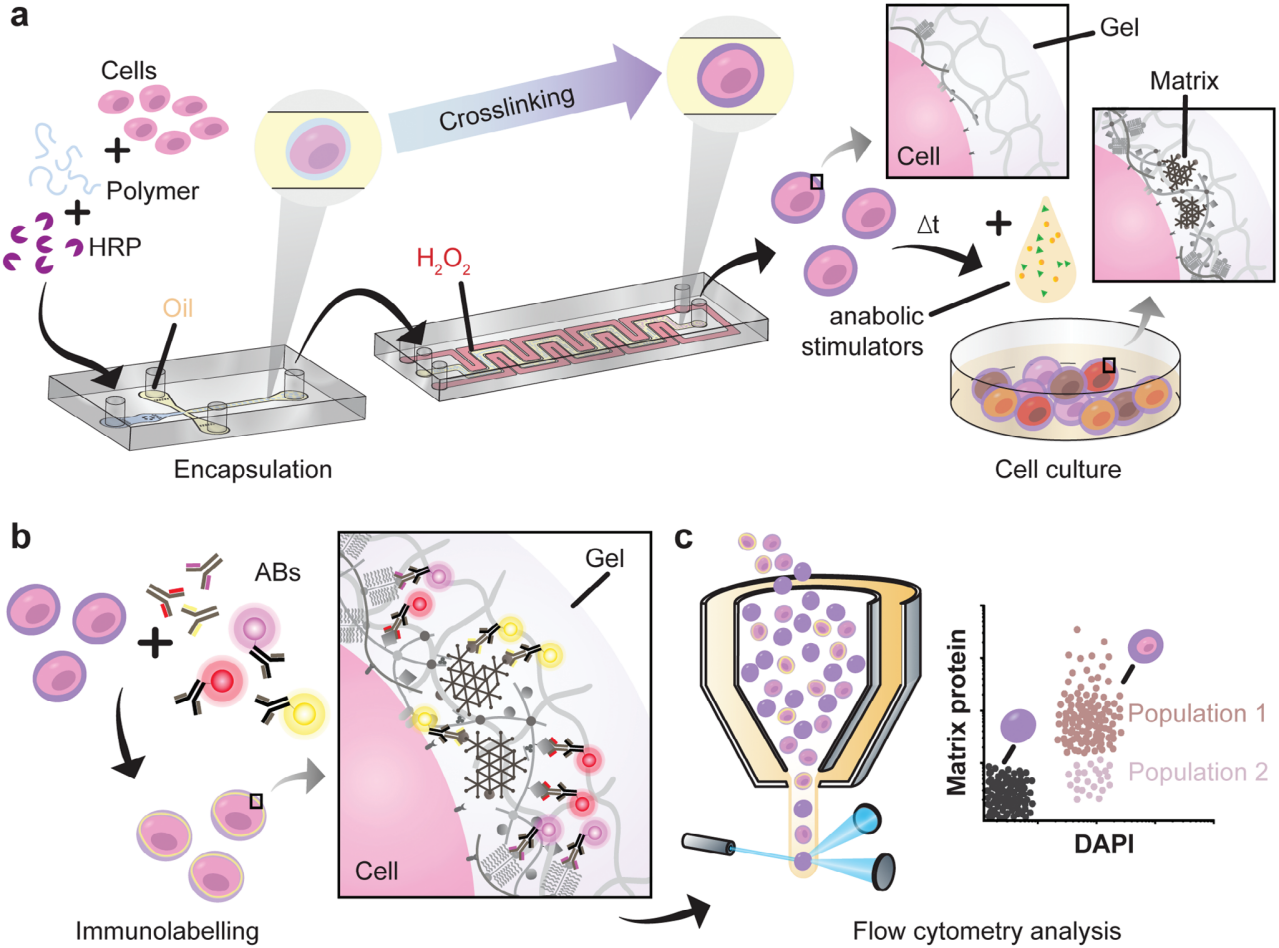
Schematic overview of the principles and workflow of Extracellular Protein Identification Cytometry (EPIC). a) Microfluidic encapsulation of individual cells in microgels allows for matrix deposition inside a 3D tunable engineered microniche. b) Deposited nascent ECM proteins of interest can be fluorescently immunolabeled, c) Flow cytometry analysis in high‐throughput for single cell (semi‐) quantitative analysis and subpopulation identification.

To engineer 3D single‐cell microniches in which cells can deposit their nascent ECM, an advanced microfluidic platform was used (**Figure** **1**a–c). Tyramine‐functionalized Dextran (Dex‐TA) was selected as the model biomaterial to engineer EPIC's microniches owing to its high cytocompatibility, chondrogenic conducive nature, and versatile tyramine chemistry (i.e., oxidative phenolic crosslinking).^[^ ^15^ ^]^ Specifically, oxidative phenolic coupling enables mechanotransduction via discrete inducible on‐cell crosslinking, offers mechanical tunability of the hydrogel (e.g., stiffness of the hydrogel), allows for bio‐orthogonal post‐crosslinking, and is compatible with immunohistological analysis as well as microfluidic cell encapsulation.^[^ ^16^ ^]^ Moreover, the oxidative phenolic crosslinking approach is near‐universal as a model system, as it is compatible with a wide variety of polymer backbones. As a cell model, human primary chondrocytes (hPCs) were selected because matrix deposition and matrix remodeling are key functional biomarkers of proper chondrogenic function (e.g., homeostasis) as well as pathological dysfunction (e.g., osteoarthritis).^[^ ^17^ ^]^ Notably, Dex‐TA has been shown to support chondrogenic behavior,^[^ ^15b^ ^]^ and is currently used in a phase 1 human trial to treat cartilage defects.^[^ ^18^ ^]^ Hence, it was chosen as a clinically relevant model system.

**Figure 2:**
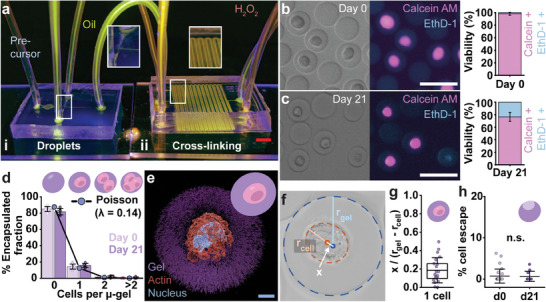
Centered single‐cell microencapsulation for long‐term cell culture within an engineered 3D microniche. a) Fluorescent photograph of two in‐line connected microfluidic devices used for cell encapsulation: i) Droplet generator in flow focus mode that is in‐line connected with a ii) delayed crosslinking chip that contains H_2_O_2_ channels that run in parallel with the droplet‐in‐oil emulsion channel, which facilitates crosslinking via diffusion of the H_2_O_2_ through the PDMS walls and the oil phase. b,c) Brightfield and fluorescent micrographs of generated single‐cell microgels stained using Calcein AM (live; pink) and Ethidium homodimer‐1 (EthD‐1) (dead; blue) after 0 and 21 days of culture and corresponding semi‐quantification (*n* = 10 images each; *n* = 294 (d0), *n* = 302 (d21) encap. cells), d) Semi‐quantification of encapsulated fractions on day 0 and day 21 post‐encapsulation, being 85.1%, 14.3%, 0.45% and 0.1% on day 0, and 81.9%, 15.9%, 1.7% and 0.55% after 21 days for empty, 1 cells, 2 cells and >2 cells respectively (n = 10 images each; *n* = 2127 (d0), *n* = 1659 (d21) µ‐gels). e) 3D reconstruction based on confocal fluorescent micrographs of a single‐cell microgel stained with EthD‐1 (gel; purple), phalloidin (actin; red) and DAPI (nucleus; blue). f) Brightfield micrograph of single‐cell microgel containing a schematic display of the parameters used to calculate cell centeredness. g) Image‐based quantification of cell centeredness of encapsulated single‐cell microgels (*n* = 21 encap. cells). h) Image‐based quantification of cell egression after encapsulation following 0 and 21 days of culture (based on 875 and 360 encap. cells, *n* = 29 (d0) and *n* = 11 (d21) images, respectively). Statistics: b–d,g,h) Error bars show the standard deviation. Bar graphs and the line in the box plots indicates the mean. Boxes indicates the 25th and 75th percentile. h) Mann–Whitney U‐test (two‐sided), n.s. nonsignificant. Scale bars: white 50 µm, blue 5 µm, red 3 mm.

To produce thin‐shelled, conformal, and stable, Dex‐TA microgels containing a single chondrocyte, we employed two in‐line connected microfluidic devices. Specifically, an in‐house developed water‐in‐oil droplet generator, that was operated in flow focus mode, was used to produce droplets composed of a 5% Dex‐TA solution containing cells and horseradish peroxidase (HRP) (**Figure** **2**a‐i). Subsequently, the resulting emulsion was continuously flown through a second microfluidic device that facilitated cell‐centering via its serpentine channel design, and delayed enzymatic oxidative phenolic crosslinking of the polymer solution droplets.^[^ ^13b^ ^]^ The latter was achieved via controlled diffusion of hydrogen peroxide (H_2_O_2_) through thin PDMS walls and the emulsion's oil phase (Figure 2a‐ii). This reaction, where HRP acted as a catalyst and H_2_O_2_ as an oxidizer, proved highly cytocompatible as confirmed by high cell viability (>95%) (Figure 2b; Figure S1a–c, Supporting information). Moreover, a high level of cell viability was maintained for at least 21 days, which represents a sufficiently long window of time to study nascent ECM protein deposition (Figure 2c).

To obtain the purest single‐cell population based on random encapsulation, we defined our Poisson distribution by a lambda of 0.14 (Figure 2d), which corresponds to a cell concentration of 10^6^ cells mL^−1^ for microgels with a diameter of 30 µm. The microgels were produced at kHz rates. By performing multiple cell encapsulations using distinct sets of microfluidic chips each time, we demonstrated the reproducibility of EPIC's encapsulation process (Figure S1d–f, Supporting information). Following the delayed crosslinking, 3D reconstruction of confocal images confirmed that each cell was coated in a 8 µm thin layer of Dex‐TA in a conformal (Figure 2e) and centered manner (Figure 2f,g). Cell centering is recognized as being essential to minimizing cell egression during cell culture.^[^ ^13b^ ^]^ Indeed, our centered single chondrocyte microgels associated with unprecedentedly low levels of cell egression (>5%) immediately after cell encapsulation, which remained stable over time (Figure 2h). It is of note that cell proliferation within the single‐cell microgels was negligible during this period. This resulted in the produced single‐cell population being stably maintained for at least 21 days. Notably, this lack of cell proliferation was not defined by the encapsulation procedure nor by the microgel's biomaterial composition, but rather by the use of chondrogenic differentiation medium as control cultures in proliferation medium were characterized by substantial levels of cell proliferation, resulting in multicell microgels (Figure S1h–k, Supporting information). Of note, chondrocytes in mature cartilage are known for their exceedingly low proliferative rate,^[^ ^19^ ^]^ a characteristic we achieved in our single cell microgels via exposure to chondrogenic medium. Taken together, this demonstrated the cytocompatible production of single‐cell microgel populations, which remain stable and viable during long‐term cell culture under nascent ECM deposition stimulating culture conditions.

### 2.2 Matrix Content Inside 3D Microniches Scales Linearly with Flow Cytometry Signal

**Figure 3:**
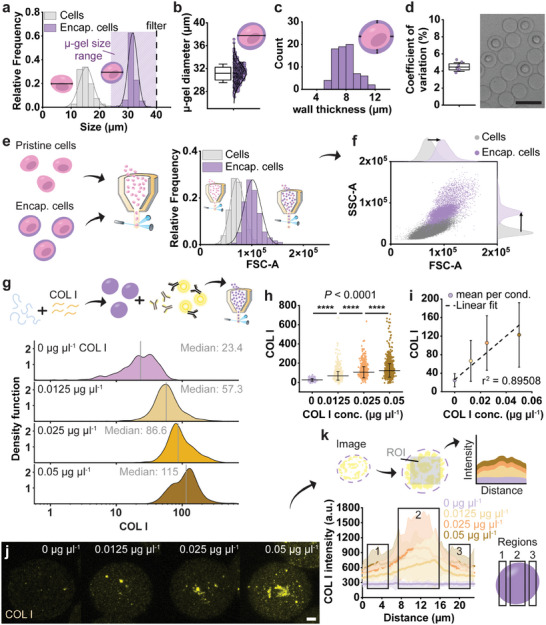
Concentration sensitive detection of COL I in cell‐sized microgels using EPIC. a) Semi‐quantification of diameter of pristine cells and encapsulated cells, showing the microgel size range (purple shaded area) being compatible with cell straining (filter) and thus clog‐free flow cytometry (*n* = 3525 cells, n = 227 encap. cells). b) Diameter of (cell‐laden) microgels giving average (line) and SD (error bars) (*n* = 715 gels), c) distribution of microgel wall thickness of encapsulated cells (*n* = 76 measurements), d) coefficient of variation for microgel diameter with mean (line) and SD (error bars) (averages from 8 images, n = 648 gels), and brightfield image showing monodisperse gels. Flow cytometry analysis of pristine and encapsulated cells: e) showing differences in forward scatter, f) forward scatter (FSC‐A) plotted against side scatter (SSD‐A), revealing increase in both scatter signals for the encapsulated cells (arrows), (*n* = 56 171 cells, *n* = 7527 encap. cells). g) Dex‐TA microgels spiked with various concentrations of COL I protein (0, 0.0125, 0.025, or 0.05 µg µL^−1^), immunolabelled with COL I antibodies and analyzed using flow cytometry. Histograms showing increase in COL I signal with increasing COL I concentration. Grey line indicates the median signal. h) Respective scatter plots displaying COL I signal distribution per COL I concentration including average (line) and SD (error bars), i) Mean COL I signal (circle) and SD (error bars) plotted against respective COL I concentration and linearly fitted with an adjusted r^2^ of 0.895, (*n* = 298 (0 µg µL^−1^), *n* = 1434 (0.0125 µg µL^−1^), *n* = 939 (0.025 µg µL^−1^), *n* = 1915 (0.05 µg µL^−1^) µ‐gels per condition). j) Maximum intensity projection (MIP) images based on confocal fluorescent micrographs (z‐stacks) of COL I spiked Dex‐TA microgels, k) Workflow of image analysis: a rectangular ROI was used, as visualized, to measure the COL I signal intensity inside the microgels using the MIP images. COL I mean intensity over the distance of the ROI (main line) with SD (shaded area) is plotted. COL I intensity is highest in the core region (2) of the microgels for COL I spiked microgels, (*n* = 42 (0 µg µL^−1^), *n* = 57 (0.0125 µg µL^−1^), *n* = 63 (0.025 µg µL^−1^), *n* = 61 (0.05 µg µL^−1^) µ‐gels per condition). Statistics: error bars indicate SD, b,d,c) boxes indicates 25th and 75th percentile, line indicates mean, h) Kruskal–Wallis test with Dunn's post hoc test, ^****^ *p* < 0.0001. Scale bars: white 5 µm, black 50 µm.

While a few pioneering studies have demonstrated that (multi) cell‐laden microgels can be flown through a flow cytometer,^[^ ^13^, ^20^ ^]^ the use of relatively large (e.g., >100 µm) microgels has been associated with low throughput due to clogging caused by their size. To prevent clogging of the nozzle during flow cytometry or fluorescence activated cell sorting (FACS), it is common practice to strain cell suspensions using a 40 µm cell strainer prior to analysis. Consequently, this sets the maximum size for the microgels to 40 µm to ensure smooth operation of the flow cytometer with minimal clogging. Advantageously, the single cell microgels are within this narrow size range (31.17 ± 1.62 µm) owing to the micrometer‐thin (7.94 ± 1.42 µm) nature of the hydrogel shell in combination with the monodisperse (coefficient of variation of 4.46% ± 0.51%) microfluidic droplet generation (**Figure** **3**a–d). This ensured that the entire (single‐cell) microgel population was uniform in size, thus making it directly compatible with conventional flow cytometers in a clog‐free manner. Microencapsulation increased both forward and side scatter as compared to pristine cells, which makes the single cell microgels an identifiably distinct population allowing for label‐free identification of cell‐laden microgels using flow cytometry (Figure 3e,f; Figure S2a, Supporting information). We demonstrated that the produced single‐cell microgels were consistently small enough to be directly analyzed using a conventional flow cytometer in high‐throughout, without the need for any cell retrieval step, which thus allows to retain the spatial and cellular information of the microniche, and paves the way for in situ nascent ECM analysis.

To test whether matrix proteins within the microgels could be detected by flow cytometry, Dex‐TA microgels were spiked with exogenous human collagen type I (COL I; Figure 3g). We created Dex‐TA microgels containing COL I at three different concentrations (0.0125, 0.025, and 0.05 µg µL^−1^) to determine whether differences in matrix concentration lead to proportional differences in labeling signal. Advantageously, phenolic moieties, such as TA, can covalently bond via enzymatic oxidative crosslinking with numerous ECM components including COL I by reacting with the matrix protein native phenolic moieties (e.g., tyrosine), as previously reported.^[^ ^21^ ^]^ To verify this in our system, we stained COL I spiked microgels either directly after retrieval, or following incubation in PBS for three days, to allow for COL I diffusion out of the microgels. Confocal imaging confirmed that COL I proteins were successfully immobilized within the microgels, as no decrease in COL I signal intensity was observed over time (Figure S2b, Supporting information). In line with this observation, the flow cytometry signal of fluorescently immunolabeled COL I containing microgels scaled linearly to the COL I concentration (Figure 3g–I; Figure S2c, Supporting information). While offering a linear signal on average, we also noted that COL I spiked Dex‐TA microgels were associated with an observed heterogeneity in fluorescent (COL I) signal. Confocal microscopic investigation confirmed that COL I, although present in all microgels, was incorporated in a globular and heterogeneous manner (Figure 3j). Further image analysis confirmed both the COL I concentration‐dependent proportional increase in mean fluorescent COL I signal, and the increased intra‐bead heterogeneity in the COL I spiked microgels (Figure 3k; Figure S2d, Supporting information). Of note, natural nascent ECM deposition also typically occurs in a spatially heterogeneous manner throughout the hydrogel.^[^ ^22^ ^]^ As such, the COL I protein agglomerates, though unintended, allowed us to more accurately mimic nascent ECM deposition. Notably, EPIC enabled us to detect spiked‐in COL I at concentrations as low as 0.0125 µg µL^−1^ (≈0.2 pg per microgel), which is significantly lower than the detection range of conventionally employed bulk glycosaminoglycan (GAG) assays, hydroxyproline assays (>1 µg of proteins) or western blots (>0.1 ng of proteins). Together, this confirmed that using EPIC, fluorescently stained ECM could be (semi‐)quantified in our engineered microniches in a sensitive, linear, and straightforward manner.

### 2.3 EPIC Enables High‐Throughput Single‐Cell Analysis of Labeled ECM Proteins

**Figure 4:**
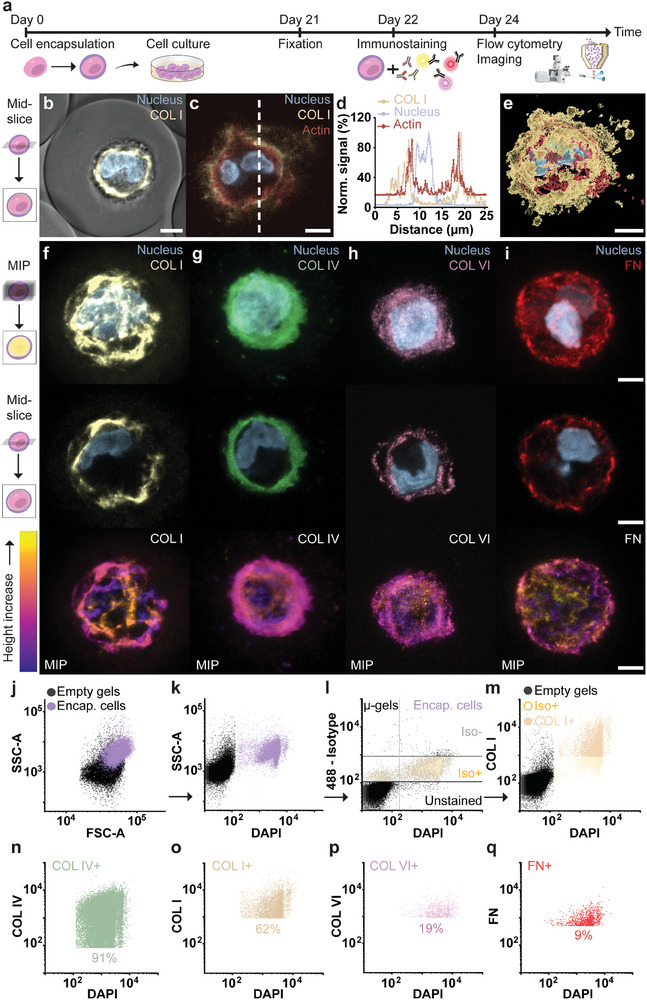
EPIC reveals heterogeneity in deposited nascent ECM proteins. a) Schematic time line for nascent ECM deposition and analysis using EPIC: hPCs are cultured in single‐cell microgels in chondrogenic medium for three weeks, followed by cell fixation, fluorescence immunostaining, and confocal and flow cytometry analysis. b) Confocal mid‐sagittal micrograph of single hPC microgel stained for DAPI (nucleus) and COL I, c) confocal mid‐sagittal micrograph of single hPC microgel stained with DAPI (nucleus), phalloidin (actin), and COL I. Dotted white line indicates where an intensity analysis was done, c) which is depicted in corresponding histogram. e) 3D reconstruction of confocal micrographs of single hPC microgel stained for nucleus (blue), cytoskeleton (red), and COL I (yellow). f)‐i) Immunostaining of encapsulated hPCs for COL I (yellow), COL IV (green), COL VI (pink), and FN (red) with nuclear counterstaining (blue). Panel displays per condition a confocal z‐stack based MIP, mid‐section, and spatial color coding of the respective antibody signal (MIP), reading from top to bottom. Flow cytometry data of immunolabelled encapsulated hPCs showing the gating procedure used for EPIC: j) forward‐ and side scatter plot showing differences for encapsulated and pristine cells, k) DAPI staining allows to distinguish the pristine microgels from cell‐laden microgels, l) signal of isotype control sample in 488 and DAPI channel, included to exclude signal that could arise from aspecific antibody binding, m) signal of COL I stained encapsulated cells in 488 and DAPI channel, showing gating for the COL I+ population. Showing flow cytometry data of encapsulated hPCs gated as explained above, for n) COL IV+ (*n* = 80 053 microgels), o) COL I+ (*n* = 69 946 microgels), p) COLVI+ (*n* = 44 289 microgels), and q) FN+ (*n* = 97 427 microgels). Percentage in the flow cytometry graphs indicates the percentage of single hPC‐laden microgels positive for each respective stain. Scale bars: 5 µm. Legend: iso – isotype control positive (+) or negative (−) population, encap. cells – encapsulated cells, COL – collagen, FN – fibronectin, MIP – maximum intensity projection.

To investigate whether EPIC also enables the quantification of cell‐secreted and thereafter deposited ECM proteins (i.e., mature ECM proteins), we microfluidically encapsulated hPCs in microgels, and cultured the resulting single‐cell microgels for up to three weeks, to allow for matrix deposition (**Figure** **4**a). As a proof of principle, we used antibodies to stain cultured encapsulated cells for COL I, which is a well‐known biomarker for chondrocyte dedifferentiation and cartilage fibrosis.^[^ ^23^ ^]^ Confocal microscopy confirmed that COL I was deposited within the microgels in the cell's pericellular domain (Figure 4b). Moreover, subsequent co‐staining of the cytoskeleton (e.g., actin) suggested that the stained matrix was indeed located extracellularly (Figure 4c–e).

Having confirmed the successful deposition and staining of nascent COL I, we optimized the antibody staining for other pericellular matrix molecules of hPCs: collagen type IV (COL‐IV),^[^ ^24^ ^]^ collagen type VI (COL‐VI),^[^ ^25^ ^]^ and fibronectin (FN)^[^ ^2^, ^26^ ^]^ using human subchondral bone tissue sections, cryo‐sectioned micromass pellets of hPCs, and single hPC microgels (Figure 4f–I; Figures S3a–d and S4a–d, Supporting information). Confocal microscopy analysis revealed that the deposited matrix was mainly located in the direct vicinity of a cell. For a limited amount of single cell microgels we also observed COL I and COL IV staining closer to the microgel's outer shell (Figure 4f,g; Figure S5a–d, Supporting information), which indicated that the cell‐deposited matrix can diffuse into the 5% Dex‐TA microgel. Together this demonstrated, that the spatial and structural information of the matrix of individual cells is retained inside an isolated, fully 3D, microniche that can be physically handled, chemically manipulated, and studied with techniques such as confocal microscopy.

We proceeded with flow cytometry analysis of single‐cell microgels stained for COL I, COL IV, COL VI, or FN. Importantly, the use of microgels allowed the analysis to be performed without the need for physical or enzymatic matrix destruction to facilitate cell retrieval. DAPI staining enabled a clear and complete separation of the encapsulated cell population compared to pristine (i.e., cell‐free) microgels (Figure 4j,k; Figure S6a–k, Supporting information). This allowed for an accurate selection of cell‐laden microgels while ignoring empty microgels. Nonspecific binding of matrix‐targeting antibodies was determined and normalized using antibody isotype controls for different matrix‐targeting antibodies (Figure 4l,m). Flow cytometry analysis of immunostained microgels revealed a large inter‐ECM protein variation in deposited nascent matrix levels. While nearly all encapsulated hPCs deposited COL IV (91%; Figure 4n), approximately two‐thirds of the hPC population stained positive for COL I (66%; Figure 4o), and only one‐fifth of the population was characterized by detectable COL VI deposition (19%; Figure 4p). Few encapsulated cells showed a fibronectin signal higher than the respective isotype control sample (9%; Figure 4q). As an additional control, we also analyzed any remaining matrix deposition on hPCs following trypsinization, which revealed that most but not all ECM proteins were removed, with a notable inter‐cell heterogeneity for some ECM components (Figure S7a–d, Supporting information). Regardless, a clear increase in ECM proteins was detected for all tested ECM components after three weeks of culture. Owing to the single‐cell microgel approach, EPIC also allowed for the investigation and quantification of ECM protein heterogeneity within the analyzed cell populations. This revealed a large spread in labeled deposited nascent matrix within the population. For example, variation in COL I deposition between distinct single hPC microgels spanned more than one order of magnitude (10^3^ to more than 10^4^) (Figure 4o; Figure S8a–d, Supporting information). Notably, the measured deposited matrix inside the microgels not only depends on the matrix production (e.g., such as pro‐collagens), but also on the secretion rate of matrix processing enzymes (e.g., proteases) and matrix degrading enzymes (e.g., matrix metalloproteinases). Hence, a variety of distinct factors jointly contribute to an inter‐cell heterogeneity in matrix deposition. Given the nanoporous nature of the hydrogel,^[^ ^27^ ^]^ cell‐secreted pro‐peptide molecules (e.g., pro‐collagens) can potentially diffuse out of the microgels when not naturally crosslinked. To verify this, pure Dex‐TA microgels were laden with COL I protein solution via diffusion overnight, and stained either directly or following an incubation in a PBS bath for three days. Confocal imaging revealed that virtually all COL I had diffused out of the microgels after three days (Figure S2e, Supporting information), thus corroborating that secreted noncrosslinked matrix can diffuse efficiently out of the microgels. Therefore, the matrix signal measured using EPIC reports on matrix that is both secreted and deposited (i.e., mature matrix proteins). It is of particular significance that our engineered monodisperse microniches offer (near) identical conditions (including diffusion) to all encapsulated cells. Hence, this enables us to provide quantitative data on single cell matrix deposition. In contrast, in bulk hydrogels the properties of the microenvironment of embedded cells depends on the cell's spatial placement (e.g., in the center of the bulk hydrogel, or in its periphery) due to differences in diffusion distances.^[^ ^28^ ^]^ While matrix deposition has been reported to be heterogeneous in bulk hydrogels,^[^ ^29^ ^]^ so far this analysis was limited to small cell numbers, and inherently biased due to the nonhomogenous nature (i.e., in terms of diffusion differences) of the bulk hydrogel. Thus, EPIC provides a 3D hydrogel environment that avoids traditional drawbacks of bulk hydrogels. To visualize the heterogeneity in chondrocyte matrix deposition in bulk hydrogels (i.e., excluding that the phenomenon is microgel specific), we cultured hPCs in a bulk hydrogel and metabolically labelled the nascent matrix proteins (Figure S9a–d, Supporting information). This corroborated that also in bulk hydrogel the hPCs show a large variety in matrix deposition. Taken together, we demonstrated that EPIC can reveal cell‐deposited inter‐ and intra‐ECM protein variation. Unlike image analysis, the use of flow cytometry enabled us to analyze >10^5^ cells in a matter of minutes, a feat that allowed us to identify and distinguish cellular subpopulations that differ in their quantity of matrix deposition. Finally, to the best of our knowledge, we here demonstrated for the first time the in situ analysis of immunolabeled deposited ECM proteins at the single‐cell level in high‐throughput, utilizing flow cytometry. While it goes beyond the scope of this work, we anticipate that this also offers novel opportunities to study the effect of various cell‐material interactions on matrix deposition based on altered material formulations such as variations in stiffness, stress relaxation, degradation rate and binding moieties among others. Of note, although Dex‐TA was used as a model material in this study, the microfluidic encapsulation system is readily compatible with a wide variety of biomaterials, as it is compatible with photo‐induced, enzymatic, ionic, and click‐chemistry crosslinking, among others, as well as the use of other cell types and various hydrogel formulations.^[^ ^13^, ^16^, ^21^ ^]^ Due to the use of oxidative phenolic crosslinking, biological factors such as growth factors or cell binding moieties (e.g., RGDs, NVADs) could also be incorporated in the engineered microniches to orchestrate desired biological responses.^[^ ^21a^ ^]^ Furthermore, we also envision that on‐demand matrix capturing of moieties is possibly via on‐demand crosslinking, which effectively bonds the microgels’ tyramines to the tyrosine's naturally present in matrix proteins. For ECM proteins, antibodies are currently the most prevalently used, versatile, and commercially available binding moieties, we therefore opted for their use as our model binding moiety. However, the staining procedure of the microgels is not limited to antibodies as EPIC is also inherently compatible with alternative binding moieties, such as aptamers, peptides, and nanobodies. Additionally, while we focused on flow cytometry analysis, the omission of the cell retrieval step made possible by the engineered 3D microniche, may also open up new possibility for performing single cell ECM analysis employing mass cytometry or potentially even mass spectrometry.

### 2.4 EPIC Allows for the Detection of Changes in Matrix Deposition Frequency and Intensity

**Figure 5:**
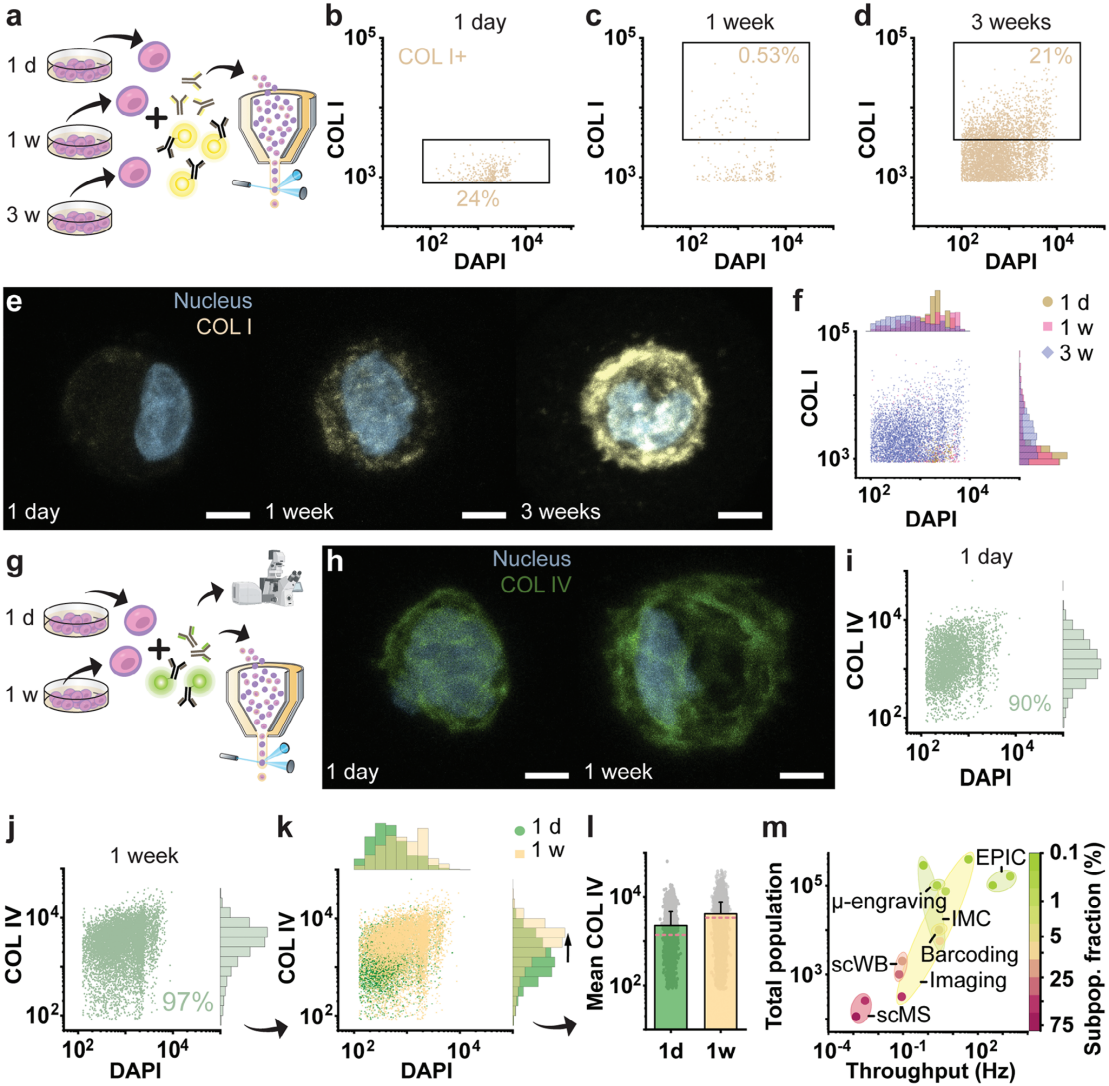
EPIC allows for early detection of nascent ECM deposition at the single‐cell level in high‐throughput. a) Schematic workflow for time‐resolved analysis of COL I deposition using EPIC: encapsulated cells are culture for 1 day (1d), 1 week (1w), or 3 weeks (3w). Respective flow cytometry data of COL I (yellow) and DAPI (blue, nuclei) stained encapsulated single hPCs after b) 1 day (*n* = 5069 microgels), c) 1 week (*n* = 91 345 microgels), d) 3 weeks (*n* = 50 182 microgels) of culture. The first one shows the percentage of COL I+ encapsulated cells and gate (black rectangle) for day 1 COL I+ levels. The latter two include the gate (black rectangle) and percentage for the COL I+ population above day 1 levels. e) Corresponding representative images of the increase in COL I deposition inside the microgels over time, showing deposited COL I (yellow) and nuclei (blue) of the encapsulated cells. f) Overlay of flow cytometry data (COL I & DAPI+) of all three time points, including histograms showing the population distribution. g) Schematic workflow for time‐resolved analysis of COL IV deposition after 1 day and 1 week of culture. h) Confocal MIP images depicting deposited COL IV (green) and nuclei (blue) of encapsulated single hPCs after 1 day and 1 week. Respective flow cytometry data of COL IV and nuclei stained single encapsulated hPCs after i) 1 day (*n* = 4155 encap. cells) and j) 1 week (*n* = 7929 encap. cells), including histograms displaying the signal distribution of COL IV. k) Overlay of flow cytometry data of different time points, showing increase in COL IV signal intensity over time (arrow). l) Bar graph comparing mean COL IV intensity, and median intensity (pink dotted line) for the two different time points, with overlay of single cell data points (grey dots). Error bars indicate SD. m) Comparing throughput of different proteomic analysis techniques (potentially) compatible with ECM analysis of single cells, including single cell mass spectroscopy (scMS),^[^ ^7^, ^32^ ^]^ automated imaging (imaging time only),^[^ ^33^ ^]^ single cell western blot (scWB),^[^ ^9^, ^34^ ^]^ microfluidic DNA‐barcoding of antibodies (barcoding time only),^[^ ^35^ ^]^ imaging mass cytometry (IMC),^[^ ^10^, ^36^ ^]^ µ‐engraving (an microarray technique)^[^ ^8a^ ^]^ and EPIC (flow cytometry throughput). For calculation of the smallest subpopulation fraction identifiable, indicated with the color code, a CV <5 was assumed.^[^ ^37^ ^]^ Scale bars: 5 µm.

As EPIC benefits from the analytical power of flow cytometry, we hypothesized that time‐resolved analysis could be leveraged to gain insights into the matrix deposition rates within cell populations over time to identify early/high performing cells. To this end, we microencapsulated individual hPCs in microgels, exposed them to chondrogenic differentiation medium, and analyzed the deposited ECM proteins after one day, one week, or three weeks of culture (**Figure** **5**a). Analysis of COL I deposition revealed that after one day, a small fraction of hPCs was already positive for COL I. Gating was used to control for incomplete removal of the matrix from freshly encapsulated hPCs (Figure 5b; Figure S7a, Supporting information). This approach revealed a cellular subpopulation (0.53%) depositing detectable amounts of nascent COL I as soon as 1‐week post‐encapsulation (Figure 5c). Confocal microscopy corroborated that a subset of single hPC microgels had detectable levels of COL I, which suggested that EPIC could indeed accurately identify and quantify early high performers within a large population (Figure S10a–c, Supporting information). After being cultured for three weeks, it was determined that 21% of encapsulated hPCs had deposited detectable levels of COL I above day one levels (Figure 5d). This demonstrated that COL I deposition was heterogeneous within the hPC population and increased in frequency over time (Figure 5e), highlighting that time‐resolved EPIC analysis can be used to map the deposition rates of nascent ECM on the single‐cell level in an accurate, quantitative, and high‐throughput manner.

Next, we investigated whether EPIC could also be used for the accurate quantification of nascent ECM proteins that are either rapidly deposited at high levels following enzymatic retrieval or are still high after enzymatic retrieval due to incomplete removal, such as COL IV (Figure 5f; Figure S7b, Supporting information). Confocal imaging confirmed that COL IV was already present in detectable levels for the majority of cells after a single day of culture (Figure 5g). As a control, we also analyzed hPCs following trypsinization (i.e., that is without encapsulation), which revealed that not all COL IV could be successfully removed (Figure S7b, Supporting information). Hence, the high COL IV levels on day one, were likely the result of partial COL IV removal. Unlike for COL I deposition, the differences in matrix deposition of COL IV, comparing microgels of one day and one week post‐encapsulation, were less easily distinguished (Figure 5g,h). Despite the early upregulation and less distinct differences, EPIC enabled us to detect changes in COL IV deposition after just one week of culture in both population frequency (90% after 1 day to 97% after 1 week) as well as intensity (Figure 5i–k). While the shift in population frequency was small, there was a distinct increase in population (median) intensity (Figure 5k,l). Notably, the single‐cell data revealed that a small part of the population deposited COL IV at low levels at both time points, which did not seem to increase over time. In comparison, conventional bulk data presentation only shows a small shift in population average (Figure 5l). This shift would likely be interpreted as an increase in matrix deposition per cell, obscuring the heterogeneity within the population.^[^ ^30^ ^]^ This illustrates how single‐cell information on matrix deposition as provided by time‐resolved EPIC analysis can help detect increases in the matrix deposition (i.e., staining intensity) that would otherwise be difficult to quantify. Taken together, it also demonstrates that EPIC is particularly suitable for the early detection of nascent matrix deposition of individual cells. Similar to other assays employing antibodies, the linear detection range of EPIC is dependent on the quality of the chosen antibody. We envision that EPIC can also be used for the detection of matrix deposition at the end‐stage of tissue formation (e.g., approximating matrix levels akin to native mature tissues), however, this may require further optimization of the protocol to achieve a suitable signal to noise ratio (i.e., to avoid measuring above saturation levels), which is essential to avoid technical variation to be a signal contributor and thus to accurately measure inter‐cellular heterogeneity.

As EPIC is inherently compatible with FACS, isolation of subpopulations could be readily achieved via sorting for further downstream analyses. Indeed, compared to current (ECM) protein analysis techniques, EPIC paves the way for the identification of small subpopulations, given the unprecedented high‐throughput nature of the technology, and thus the ability to analyze large populations within reasonable time frames (Figure 5m; Figure S10d, Supporting Information). Notably, the throughput of the cell encapsulation process occurred at ≈2000 microgels s^−1^, thus confirming with the high‐throughput nature. The detection and analysis of subpopulations associated with altered matrix deposition or remodeling are anticipated to aid elucidation of molecular mechanisms governing pathologies, determine drug efficacies, contribute to personalized medicine, and improve stem cell differentiation protocols. To elaborate on potential downstream analyses, isolated subpopulations of interest can be readily analyzed with virtually any imaging technology (e.g., confocal imaging, holotomography, TEM, nanoCT), if desired in combination with prior histological preparation (e.g., mechanical sectioning and staining). Also, downstream proteomics analysis of isolated populations (e.g., mass spectrometry and spatial proteomics), gene expression analysis (e.g., single cell RNA sequencing^[^ ^31^ ^]^) or metabolic investigation (e.g., metabolic flux analysis) are anticipated to be inherently compatible with EPIC. Of note, if necessary, the hydrogel layer surrounding the cells can be removed post‐sorting by using bio‐orthogonal enzymes such as dextranase. Moreover, EPIC can potentially be combined with live‐cell staining procedures as it relies on the labeling of extracellular proteins, thus in principle omitting the need for cellular fixation and permeabilization.

### 2.5 Nascent Matrix Deposition Is Dependent on Cell–Cell Contact

**Figure 6:**
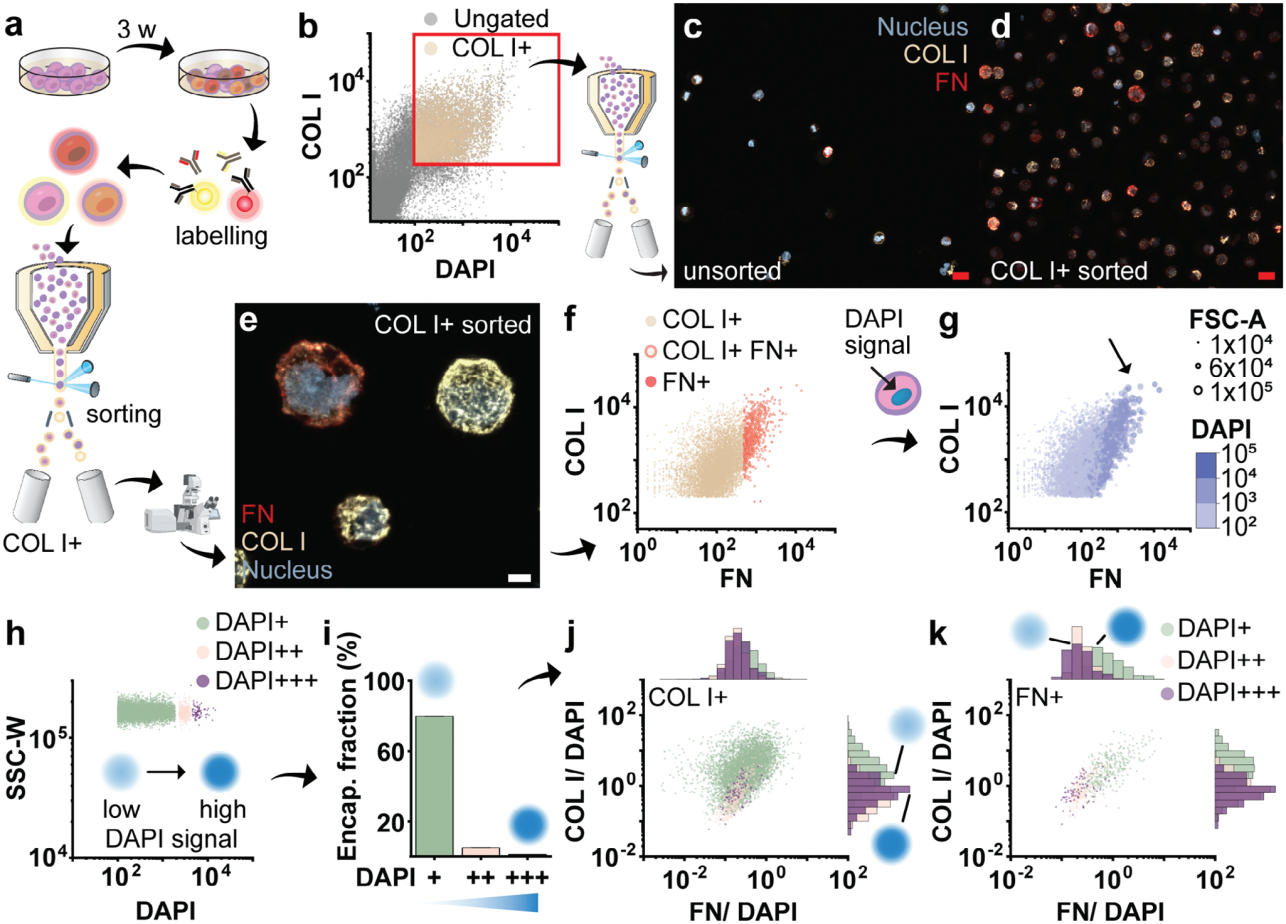
EPIC is compatible with multiplexed analysis of deposited nascent ECM. a) Workflow for multiplex analysis using EPIC: cell culture for three weeks, followed by antibody staining for COL I and FN, and flow cytometry analysis, combined with FACS and downstream confocal imaging of sorted populations. b) Scatter plot of COL I and FN stained encapsulated hPCs, showing DAPI signal and COL I signal. Sorted for the COL I+ population (red rectangle). c) Confocal fluorescent MIP tile scan of unsorted microgel population with nuclei (blue), COL I (yellow) and FN signal (red), d) Confocal fluorescent MIP of tile scan of COL I+ sorted encapsulated cells. e) Confocal fluorescent MIP images of COL I+ sorted microgels, showing variation in COL I (yellow) and FN (red) deposition. Flow cytometry data showing: f) Relation between COL I and FN deposition: showing the COL I+ population (yellow dots), the COL I+ FN+ population (yellow dots with red outline), and the FN+ population (red dots), g) depicting the same populations as shown in f, with the corresponding DAPI signal (indicated by blue coloring) and FSC signal (indicated by dot size). Positive correlation of higher DAPI signal and higher FSC signal with elevated matrix signal is highlighted (arrow). Encapsulated cells were gated based on DAPI signal: h) identified subpopulations of encapsulated hPCs based on DAPI intensity (DAPI+, DAPI++, DAPI+++), and i) corresponding percentages of the encapsulated cells per subpopulation. Scatter plots combined with histograms showing the distribution of the population. Plots show the COL I and FN signal normalized with the DAPI signal for: j) the COL I+ population, and the k) FN+ population. Blue dots indicate the intensity of the DAPI signal, scaling from low to high intensity for subpopulations DAPI+ to DAPI+++ respectively. Scale bars: red 20 µm, white 5 µm.

To gain deeper insights into the relation between the deposition of different ECM proteins, and to test whether EPIC is compatible with multiplex analysis, we stained encapsulated cells for both COL I and FN following three weeks of cell culture (**Figure** **6**a). FACS was used to purify COL I‐positive microgels, thereby validating the gating procedure (Figure 6b). The efficacy of this procedure was confirmed by confocal imaging (Figure 6c–e).

Plotting the COL I signal against the FN signal revealed a strong positive correlation (Figure 6f). This correlation between higher FN and higher COL I deposition is likely explained by FN's ability to bind to COL I,^[^ ^38^ ^]^ which is known to orchestrate their co‐localization.^[^ ^39^ ^]^ Moreover, while there was a substantial subpopulation of hPCs that was positive for COL I and negative for FN, it was exceedingly rare for a hPC to be positive for FN and negative for COL I. It is thus tempting to speculate that the presence of COL I indeed promoted the FN fibril formation, as has been previously suggested.^[^ ^39^ ^]^ Regardless, when also considering forward scatter (i.e., microgel size) and DAPI signal, we revealed that higher protein deposition positively correlated with DAPI signal and forward scatter (Figure 6g). Further, we noted that among the sorted cell‐laden microgels, microgels with more than one cell inside were present (Figure S11a, Supporting information). Based on this observation, we hypothesized that the population high in DAPI signal and forward scatter included multicell microgels.

We postulated that single and multicell microgels could be gated based on their DAPI signal^[^ ^40^ ^]^ to separate the populations from each other. To this end, we defined three DAPI subpopulations, which we termed DAPI+, DAPI++, and DAPI+++. We hypothesized that these represent the pure single‐cell microgel population, and populations enriched in two‐cell microgels and three‐cell microgels (i.e., multicell microgels), respectively (Figure 6h). These subpopulations respectively made up ≈80%, 5%, and 1% of the DAPI+ encapsulated population, thus closely following the predicted Poisson ratio for the cell‐laden microgel populations (Figure 6i). Flow cytometry data from the three DAPI subpopulations confirmed that both COL I and FN signals scaled with predicted cell‐occupation of the microgels (Figure S11b,c, Supporting information). Although it is logical that a higher number of cells per microgel would lead to a higher total amount of deposited nascent ECM per microgel, we investigated whether normalization of the matrix signal to the DAPI signal, to correct for cell‐occupation in individual microgels, would alter the amount of nascent ECM deposition per cell. To this end, we divided the matrix signal by the DAPI signal to estimate the per‐cell matrix deposition. Interestingly, this revealed that relative COL I and FN deposition was lower for microgels with a high DAPI signal (i.e., DAPI+++), and presumably multicell occupation, as compared to microgels with a low DAPI signal (i.e., DAPI+; single‐cell laden µ‐gels) (Figure 6j,k). Thus, suggesting that the presence of another cell in the microgel may minimize the deposition of COL I and FN per cell. Of note, the hPCs used for this study were isolated from osteoarthritic cartilage, and expanded in monolayer cell culture prior to cell encapsulation. As such, the chondrocytes were expected to show phenotypic signs of OA and chondrocyte dedifferentiation (i.e., relatively high deposition of COL I). Apart from this, it demonstrated that multiplexing of antibodies, in combination with a nuclear dye, is possible. We also envision that further multiplexing is possible (e.g., detecting multiple ECM components simultaneously) given this approach is readily compatible with flow cytometry.

**Figure 7:**
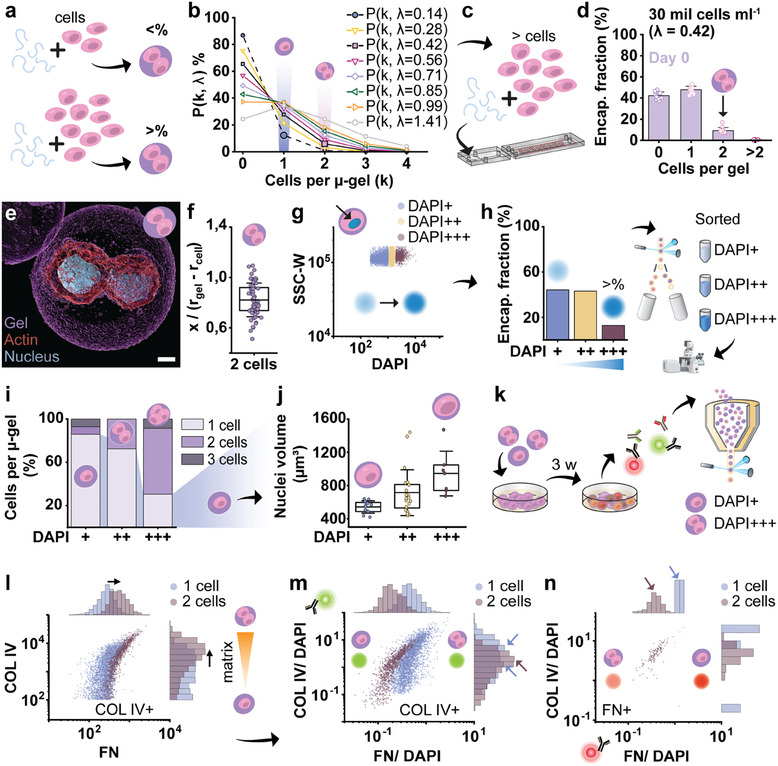
Influence of cellular co‐cultures on nascent matrix deposition as quantified by EPIC. a) Schematic showing the idea of using a higher cell density for the hydrogel precursor solution to get an enriched two‐cell laden microgel population. b) Theoretical values for the Poisson distribution of different encapsulated fractions (k = 0–4 cells per µ‐gel) and various cell concentrations (i.e., varying lambda) assuming a microgel diameter of 30 µm. c) Cell encapsulation was performed with a higher cell concentration (30 mil cells mL^−1^, corresponding to a lambda of 0.42). d) Semi‐quantification of the encapsulated fractions for the cell encapsulation with 30 mil cells mL^−1^. e) 3D reconstruction of a two‐cell laden microgel with nuclei (blue), cytoskeletal (red) and hydrogel staining (purple), and f) image‐based quantification of cell centeredness for paired encapsulated cells for the two‐cell enriched population (30 mil cells mL^−1^). g) Identification of subpopulations based on DAPI intensity for the two‐cell enriched population, with h) corresponding percentages, showing enrichment in subpopulations DAPI++ and DAPI+++. The identified subpopulations were sorted using FACS and subsequently imaged with the confocal microscope. i) Distribution of encapsulated cell fraction per DAPI subpopulation, determined based on confocal imaging and 3D reconstruction of the sorted microgels, and j) nuclear volume of cells inside single‐cell laden microgels plotted per DAPI subpopulation. Nuclear volume was obtained from the 3D reconstructions. k) The two‐cell enriched population (30 mil cells mL^−1^) was cultured for 3 weeks and stained for COL IV and FN, followed by flow cytometry analysis. Respective flow cytometry data showing: l) The relation between COL IV and FN deposition for the one cell and two‐cell sorted DAPI populations (corresponding to DAPI+ and DAPI+++ respectively), showing increased matrix deposition for the enriched two‐cell laden population, and the DAPI‐normalized COL IV and FN signal for m) the COL IV+ subpopulation, and n) the FN+ subpopulations. The large dots in the graphs (COL IV green, FN red) indicate the presence or absence of a shift in COL IV and FN intensity per cell for the single cell and two‐cell laden microgels. Arrows indicate the histograms of interest for each of the two subpopulations, COL VI+ and FN+ respectively. Statistics: d) bars indicate mean, error bars indicate SD, f,j) boxes indicates 25th and 75th percentile, line indicates mean, error bars indicate SD. Scale bar: 5 µm.

To further study the effect of cell co‐culture on nascent matrix deposition, we enriched the two‐cell population to be able to study this effect on a larger cell population, and thus take full advantage of the high‐throughput analysis. To accomplish this, we increased the encapsulation lambda by using a higher cell concentration in the hydrogel precursor solution, which enriched the fraction of microgels containing two or more cells (**Figure** **7**a,b). Specifically, the cell concentration was increased to 30 mil cells mL^−1^, which increases the two‐cell microgel population by sixfold (Figure 7c,d). Confocal imaging confirmed the conformal encapsulation of two‐cell microgels (Figure 7e), which was further supported by semi‐quantification of the cell‐centeredness of the two‐cell population (Figure 7f).

The enriched two‐cell population was then gated and sorted based on the DAPI signal (Figure 7g). As expected, the fraction of encapsulated cells in subpopulations DAPI++ and DAPI+++ drastically increased (Figure 7h). Specifically, the subpopulations DAPI++ and DAPI+++ were increased to 39% and 11% of the cell‐laden microgel population. Using FACS to sort each of the three DAPI populations, and combining it with downstream confocal imaging, we determined the number of cells per gel in each of the three subpopulations (Figure 7i). While the subpopulation DAPI+ was highly enriched in encapsulated single‐cells (85%), DAPI+++ showed a high enrichment of double‐cell‐laden microgel (61%). Concurrently, the DAPI++ subpopulation consisted of single‐cells (72%), and double‐cell‐laden microgels (28%). Given this overlap between single‐cell and double‐cell populations, we decided to assess the nuclear volume of encapsulated cells, as it is known that the DAPI signal scales with nuclear volume.^[^ ^41^ ^]^ Volume quantification of 3D‐image reconstructions of the stained nuclei corroborated that the DAPI signal increases linearly with nuclear volume (Figure 7j; Figure S11d, Supporting information). Due to the large spread in nuclear volume, the nuclear signal of two paired cells can be similar to that of one cell, explaining the overlap of those two populations in subpopulation DAPI++. Interestingly, it has been reported in literature that nuclear volume scales with cell volume.^[^ ^42^ ^]^ Thus, we can use the DAPI signal to distinguish between the different encapsulated fractions, and potentially relate the effects of cell volume to nascent ECM deposition.

Classifying DAPI+ as single‐cell, and DAPI+++ as two‐cell population, we investigated for a large cell population, whether co‐culturing of cells influenced COL IV and FN deposition of encapsulated hPCs. We cultured the double cell enriched encapsulated cells (30 mil cells mL^−1^) for three weeks in chondrogenic medium, and subsequently stained for FN and COL IV (Figure 7k). Interestingly, co‐culturing two cells in a single microgel did not have the same effect on COL IV, as previously observed for COL I and FN deposition. Specifically, while paired encapsulated cells produced higher total amounts of both COL IV and FN (Figure 7l), relative COL IV deposition was very similar for single and double encapsulated cells (Figure 7m), suggesting that COL IV deposition scaled proportionally to the nuclear signal. Normalized FN deposition was again higher for single encapsulated cells, although the majority of cells depositing FN were paired encapsulated cells (Figure 7l,n). Taken together, while cell co‐culture decreased the COL I and FN deposition per cell (Figure 6j,k), it had little effect on COL IV deposition (Figure 7m). Notably, FN deposition has been linked to enhancing initial cell‐cell contact, and was shown to reduce over time simultaneous with the onset of chondrogenic differentiation in pellet cell culture.^[^ ^43^ ^]^ Hence, it may explain why most FN was expressed by the enriched two‐cell laden microgel population, but generally found to be lower per cell in multicell microgels as compared to single cell microgels. It is noteworthy that in multicell microgels the presence of another cell may facilitate enhanced cell‐cell contact and cell–cell communication. However, it is also probable that the mechanical environment as perceived by the cell is altered, which may impact the matrix deposition as well. Jointly this suggests that the co‐culturing of cells does not have the same effect on all ECM proteins, thus allowing for the creation of chemically distinct microenvironments by encoding the presence or absence of cell‐cell communication within engineered microniches.

While it is well accepted that cell‐cell contact influences cell behavior, it has remained a significant challenge to directly study the effect of cell–cell contact on matrix deposition as it often requires a high‐throughput approach.^[^ ^44^ ^]^ Moreover, many platforms only allow to study the influence of cell‐cell contact by comparing the bulk results of different cell seeding densities with each other.^[^ ^45^ ^]^ We here now showed on the single cell level, that cell–cell contact is beneficial for a reduction in the OA/dedifferentiated chondrocyte phenotype (i.e., reduced COL I deposition per cell in multicell microgels), which highlights the sensitivity of EPIC. Furthermore, single‐cell analysis potentially allows to link spatial and cellular information, such as cell‐cell contact, to the respective cell's gene or protein expression by performing downstream transcriptomic and proteomic analysis.^[^ ^44^, ^46^ ^]^ Consequently, our findings identify EPIC as a novel enabling platform to study the influence of cell co‐culture on nascent ECM deposition.

## 3 Conclusion

We here introduce a novel technique named Extracellular Protein Identification Cytometry (EPIC) that enables the high‐throughput analysis of ECM proteins at single‐cell resolution. This innovative approach is based on the combination of advanced microfluidic production of single cell microgels, and flow cytometry. As single cell microgels inherently provide a 3D microenvironment for cells while allowing for suspension‐based culture, they do not require physical or enzymatic retrieval of cells to enable flow cytometry analysis. Consequently, owing to the nondestructive nature of this approach, spatial information within the 3D environment is retained. We demonstrated that this allows for mapping of cellular heterogeneity in matrix deposition by enabling analysis of large cell populations at the single cell level, which was used to elucidate the effect of external factors such as cell‐material or cell‐cell interactions on nascent matrix deposition. Moreover, subpopulations could be identified based on variations in matrix deposition, and subsequently isolated via integrated cell sorting. Specifically, nuclear staining was demonstrated to allow for sorting‐based purification of single cell microgels as well as distinguishing single cell microgels from multicell microgels. This allowed us to study the effect of cell‐cell contact on matrix deposition. We revealed that for hPCs, the presence of another cell in a microgel decreased relative COL I and FN deposition per cell, while only having a negligible effect on COL IV deposition. In summary, EPIC presents a novel method to analyze deposited ECM proteins of interest in high‐throughput at the single cell level by combining droplet microfluidics, widely available cross‐linkable polymers, commercially available antibodies, and conventional flow cytometers. It uniquely enables the linking of cellular information to matrix deposition at the single‐cell level for large cell populations, thus granting the identification of subpopulations. As cell‐cell and cell‐material/matrix interactions are instrumental in guiding cell behavior, including stem cell differentiation, disease progression, tissue development, and repair, EPIC represents a tool with which the influence of the cellular microenvironment on ECM deposition, and thus cell behavior, can be analyzed at the single‐cell level. Therefore, EPIC presents a platform that has the potential to offer novel deep insights required for the optimization of tissue engineering and regenerative medicine applications, such as drug screening platforms, cell‐therapy and organ‐on‐chip platforms.

## 4 Experimental Section

### Materials

Dextran (40 kDa, Pharmacosmos, Denmark), tyramine, lithium chloride, DMF (99% anhydrous), PNC, DMSO‐d_6_, peroxidase from horseradish (HRP; type VI), dialysis membrane 1 kDa MWCO (Spectra/Por), hydrogen peroxide (H_2_O_2_), tyramine, ascorbic acid 2‐phosphate (ASAP), iodixanol (OptiPrep), L‐Proline, dexamethasone, Human collagen type I (in solution; cat. CC050), Proteinase K, hyaluronidase (type I), collagenase II, calcein‐AM, ethidium homodimer‐1 (EthD‐1), Tween‐20, bovine serum albumin (BSA), dimethyl sulfoxide (DMSO), paraformaldehyde (PFA), fetal bovine serum (FBS) and sterile phosphate‐buffered saline (PBS) were purchased from Sigma–Aldrich/Merck. Recombinant human TGF‐beta‐3 protein was purchased from R&D systems. Insulin‐transferrin‐selenium (ITS, 100x) and PBS tablets were purchased from Gibco. Microscope glass slides (90° ground) for microfluidic chip bonding were purchased from Epredia. Cover slides for histological sections and Formol 4% (buffered) were purchased from VWR. Dulbecco's modified Eagle's medium (DMEM) with high glucose, penicillin/streptomycin (pen/strep), Phalloidin‐AF488, Phalloidin‐AF647 and 4,6‐diamidino‐2‐phenylindole (DAPI), trypsin‐EDTA, nonessential amino acids (NEAA), goat anti‐rabbit (H&L) IgG‐Alexa Fluor 488 (cat. 10 236 882) were purchased from Fisher Scientific/ThermoFisher. Anti‐collagen type I antibody (cat. NB600‐408) and iso‐mouse IgG2a (cat. NBP1‐96981) were purchased from Novus Biologicals. Anti‐collagen type IV antibody (cat. ab6586), anti‐collagen type VI antibody [EPR17072] (cat. ab182744), anti‐fibronectin antibody [A17] (cat. ab26245), iso‐rabbit IgG (cat. ab37415), and donkey anti‐mouse H&L IgG‐Alexa Fluor 647 (cat. ab150107) were purchased from Abcam. µ‐slide 18 well dishes were purchased from Ibidi. Cell strainers (40 µm) were purchased from Corning. Polydimethylsiloxane (PDMS, Sylgard 184) was obtained from Dow Corning. Aquapel was purchased from Bol.com. Pico‐Surf 1 (5%) in Novec 7500 Engineered Fluid, and PicoBreak 1 were purchased from Sphere Fluidics. Gastight syringes (Hamilton), fluorinated ethylene propylene tubing (FEP), and connectors were purchased from Inacom instruments. Low‐pressure syringe pumps (neMESYS) were purchased from Cetoni. Surfactant‐free fluorocarbon oil (Novec 7500 Engineered Fluid) was purchased from Fluorochem Limited.

### Cell Isolation and Expansion

Human chondrocytes (hPCs) were isolated from cartilage samples obtained from human OA patients who underwent total knee replacement surgery. No modification was made to the surgery or treatment (waste material). The use of patient material (nr. 2020–7255) was approved by the local ethical committee in Radboud (CMS), and all involved patients provided written informed consent. Cartilage pieces were obtained from nondegenerated areas of the joint and incubated with collagenase II in DMEM under agitation at 37 °C overnight. The hPCs recovered from the digestion were frozen directly using DMEM containing 20% FBS and 10% DMSO. For cell expansion, cells were cultured in growth medium consisting of DMEM high glucose supplemented with 10% v/v FBS, 1% v/v pen/strep, 0.2 mm ASAP, 0.35 mm L‐Proline, and 1x NEAA. Medium was refreshed three times a week. When reaching ≈80% confluency, cells were passaged using 0.25% Trypsin‐EDTA, and used till passage 4. Cells were maintained in culture at 37 °C and 5% CO_2_.

### Dextran Synthesis

Functionalized Dextran‐Tyramine was synthesized following the previously reported method.^[^ ^15b^ ^]^ Briefly, Dextran 40 kDa was functionalized with tyramine by using a urethane group as a linker. Initially, dextran (5 g) and lithium chloride (4 g) were transferred to a round bottom flask, and dried to remove excess moisture by repeatedly flushing with air and nitrogen. Subsequently, DMF (200 mL) was transferred through a metal cannula and the mixture was dissolved at 90 °C. After the solution became transparent, the mixture was cooled down to 2 °C before adding sublimated and powdered 4‐nitrophenyl chloroformate (PNC, 96%) stepwise to maintain the overall temperature below 0 °C. The reaction was carried out for 60 min until complete dissolution of PNC occurred, before precipitating it in excess ethanol. All reactions were carried out in an inert atmosphere unless otherwise specified. The precipitated mixture was vacuum filtered by washing it repeatedly with cold ethanol and diethyl ether. It was oven‐dried and analyzed using H^1^NMR spectroscopy. The degree of substitution (DS%), calculated as the number of conjugated PNC per 100 dextran monosaccharide rings, was determined by integrating signals from dextran (4H; δ 4.2–5.8 ppm) and conjugated PNC (4H; δ 7.4–7.7 and δ 8.2–8.5 ppm). The synthesized intermediate (Dex‐PNC) had a DS% of 28%. To initiate the Dex‐Tyramine reaction, the dried Dex‐PNC was dissolved in DMF (200 mL), followed by the addition of 2 eq molar excess of tyramine. The reaction was carried out for 60 min, and then the product was precipitated, and vacuum filtered by repeated washing in cold ethanol and diethyl ether. The resulting product was oven‐dried and characterized by H^1^NMR spectroscopy. The DS% was calculated by integrating signals from dextran (4H; δ 4.2–5.8 ppm) and conjugated tyramine (4H; δ 6.6–6.75 and 6.9–7.07), resulting in a DS% of 14% in the synthesized Dex‐TA conjugates.

### Microfluidic Chip Production

Silicon wafers for chip production were prepared at the MESA+ Institute for Nanotechnology following standard photolithography practices, based on previously reported designs.^[^ ^13b^ ^]^ Microfluidic chips were subsequently manufactured from PDMS and glass using the aforementioned soft lithography‐produced silicon wafers with prescribed chip dimensions. Briefly, PDMS precursor and curing agent (Sylgard 184) were mixed in a ratio of 10:1. The mixture was poured onto the prepared wafers, degassed, and baked at 60 °C overnight. Inlets were punched for the tubing, and subsequently, PDMS chips were bonded to microscope glass slides using plasma treatment (Cute, Femto Science, South Korea). To strengthen the bonding, the bonded chips were placed in the oven at 60 °C overnight. Prior to use, aquapel treatment was applied to the chips to create a hydrophobic coating.

### (Spiked)‐Microgel Generation

The hydrogel precursor solution for pure Dex‐TA microgels was prepared with final concentrations of 5% Dex‐TA and 21.5 U mL^−1^ HRP in PBS. To spike microgels with collagen type I, commercial human collagen type I protein, ready‐to‐use in solution, was added to the hydrogel precursor solution to reach final concentrations of 0.0125, 0.025, or 0.05 µg µL^−1^ COL I. All three solutions contained 5% Dex‐TA and 21.5 U mL^−1^ HRP. The same oil phase and H_2_O_2_ concentrations as used for cell encapsulation were employed. The hydrogel precursor and hydrogen peroxide solutions were cooled to 4 °C. Gas‐tight syringes were loaded with the different solutions, and the same flow rates as for cell encapsulation were used. The microgels were retrieved from the oil phase using Pico Break. The retrieved microgels were washed with PBS three times, before downstream analysis.

### Cell Encapsulation and Culture

To encapsulate cells, hPCs were grown until they reached ≈80% confluency. A single‐cell suspension was obtained by incubating the cells with 0.25% Trypsin‐EDTA for 5 min at 37 °C. After inactivation using complete growth medium, and thorough resuspension, cells were strained using a 40 µm cell strainer. The cell count was determined using an EVE cell counter (NanoEntek). For the hydrogel precursor solution, Dex‐TA stock of 20% (w/v) was mixed with basal medium, OptiPrep, HRP, and cell suspension to final concentrations of 5% w/v Dex‐TA, 8% v/v OptiPrep, 21.5 U mL^−1^ HRP, and 10 or 30 mil cells mL^−1^. For the oil phase, 2% Pico‐Surf was prepared by diluted 5% Pico‐Surf in 7500 Engineering fluid. The hydrogen peroxide solution was prepared with a final concentration of 1% H_2_O_2_ in MilliQ water. The respective solutions were loaded into gastight syringes. Flow rates were set to 2, 6, and 30 µL mL^−1^ as previously reported^[^ ^13b^ ^]^ for the polymer phase, oil phase, and hydrogen peroxide phase, respectively. A stereomicroscope setup (SMZ800, Nikon) with a camera (DFC400, Leica) was used for visualization of the droplet formation. The syringes containing the precursor solution, and the hydrogen peroxide solution, respectively, were kept at ≈4 °C throughout the entire cell encapsulation procedure. Encapsulated cells were retrieved using Pico‐Break solution. Following retrieval, encapsulated cells were transferred to conventional cell culture plates and incubated in growth medium at 37 °C and 5% CO_2_ overnight. The following day, encapsulated cells were washed and transferred to a new plate with either growth or chondrogenic medium. This step removed any nonencapsulated cells as these adhered to the original culture plate and thus resisted transfer to the new culture plate. The chondrogenic medium consisted of high glucose DMEM supplemented with 1% v/v Pen/Strep, 0.2 mm ASAP, 0.35 mm L‐Proline, 1x Sodium pyruvate, 1x ITS, and freshly added 10^−7^ m dexamethasone and 10 ng mL^−1^ TGFβ‐3. The medium was refreshed thrice a week. Cell culture was maintained at 37 °C and 5% CO_2_.

### Characterization of Microgels

Microgels intended for characterization were transferred to a transparent flat‐bottom 96‐cell culture well plate and imaged using a brightfield microscope (EVOS FL Imaging system microscope (ThermoFisher)). The brightfield images were analyzed with Fiji (ImageJ) to determine microgel diameter, hydrogel coating thickness, cell centeredness, monodispersity, and cell escape. For the latter, cells located at the edge of the microgels immediately after encapsulation were quantified, as these cells are most likely to egress. For the 21‐day time point, microgels with a morphology indicating cell escape (i.e., hollowed out) were quantified to assess the number of cells that had escaped.

### Analysis of Cell Viability

To assess cell viability, encapsulated cells were stained with Calcein AM (live) and Ethidium homodimer‐1 (dead) according to manufacturer's instructions. The cells were incubated in staining solution at room temperature (RT) for 15 min. Subsequently, the staining solution was removed and cells were washed with PBS. Images were captured using an EVOS fluorescent microscope (ThermoFisher) with the GFP and RFP filter cubes for Calcein AM and Ethidium homodimer, respectively. The images were analyzed using Fiji (ImageJ). The cell counter plugin was used to determine the encapsulated fractions, and perform semi‐quantification of the live‐dead staining. Ethidium homodimer signal was also used to determine the microgel diameter as it also stains the microgels. To this end, a thresholding and subsequent particle count was performed. The Calcein AM signal of stained pristine cells was used to determine the cell diameter (i.e., feret diameter) using the same approach.

### Immunostaining of Encapsulated Cells

Encapsulated cells were retrieved from the cell culture plates and washed with PBS. Fixation was performed with either freshly prepared paraformaldehyde solution (4%) or Formol (4%). Following washing, gels were permeabilized with 0.5% PBS‐Tween 20 (PBS‐T) solution under dynamic conditions at RT for 15 min. Subsequently, samples were washed with 3% BSA solution twice. After this step, an optional enzymatic treatment was performed for improved epitope retrieval, consisting of a 1:1 mixture of 0.1% w/v hyaluronidase in PBS combined with 5 µg mL^−1^ Proteinase K. Incubation at 37 °C for 15 min. Samples were washed once with PBS and two times with BSA (3%). Blocking was performed under dynamic conditions at RT for 1 h using a 3% BSA solution. Subsequently, samples were incubated with primary antibodies (ABs) or the respective rabbit or mouse isotype controls in blocking buffer at a concentration of 1:100 for all for all ECM‐targeting antibodies expect for COL VI (1:200). The isotype antibody concentration was adjusted to that of the respective antibody. Incubation under dynamic conditions at 4 °C overnight. Washings were performed once using 0.5% PBS‐T and twice with 3% BSA, one of which was incubated under dynamic conditions at RT for 2 h. Samples were incubated with the respective secondary antibodies Alexa Fluor 488 or Alexa Fluor 647 (1:300) in blocking buffer under dynamic conditions at RT for 2 h. Same washing procedure as before. As controls for flow cytometry, unstained samples and single‐stained samples were prepared. Nuclear counterstaining was performed using DAPI (1:100 in 1% BSA), incubated under dynamic conditions at RT for 30 min. For actin staining, phalloidin conjugated with either Alexa Fluor 488 or 647 was used according to the manufacturer's protocol. The samples were incubated under dynamic conditions at RT for 45 min. Samples were washed again using 3% BSA, before being stored in PBS at 4 °C till the analysis was performed.

### Immunostaining of (Spiked‐in COL I) Microgels

Spiked and pure Dex‐TA microgels were labeled for COL I using an identical procedure as was performed with the encapsulated cells. Briefly, microgels were fixated in Formol (4%), washed twice with PBS, and subsequently blocked in 3% BSA under dynamic conditions at RT for 1 h. Spiked microgels were incubated with a COL I antibody (1:100 dilution) in blocking buffer and incubated under dynamic conditions at 4 °C overnight. As controls, samples were incubated with their respective isotype control or blocking buffer as unstained control. Samples were washed once with 0.5% PBS‐T and twice with 3% BSA. The last wash was performed under dynamic conditions at RT for 2 h. Microgels were incubated with their respective secondary antibody Alexa Fluor 488 (1:300) in blocking buffer under dynamic conditions at RT for 2 h. Samples were washed using an identical washing procedure. Afterward, samples were either directly analyzed or stored in PBS at 4 °C until analysis.

### Confocal Analysis

For imaging of immunolabeled matrix, stained (cell‐laden) microgels were transferred to a µ‐slide 18‐well dish (Ibidi) for high resolution imaging. A fluorescent confocal microscope (Zeiss LSM 880) was used with a 40x or 63x water‐immersion objective for obtaining z‐stacks and tile‐scans. For z‐stacks with the 63x objective, a step size of 1 µm was used. Generation of maximum intensity projection (MIP) images, spatial color‐coding, and pseudo‐coloring was done using Fiji (ImageJ). Imaris software (Oxford instruments) was used to create 3D reconstructions, and to perform nuclear volume measurements.

### Flow Cytometry Analysis

Flow cytometry and FACS analysis was performed employing a FACS Aria 2 instrument (BD Biosciences) using a nozzle of 100 µm, and working with a sample pressure of 20 psi. Stained (cell‐laden) microgels or pristine cells were resuspended in 1.5% BSA, and passed through a 40 µm cell strainer prior to analysis. For optimal resolution and single‐microgel sorting, flow rates were adjusted to event rates of ≈400 events s^−1^. Per sample 50 000–100 000 events were collected, unless otherwise specified. The instrument was operated with BD FACSDiva (BD Biosciences) software. The obtained flow cytometry data was analyzed in MATLAB (MathWorks) using the Flow cytometer GUI^[^ ^47^ ^]^ in combination with fca_readfcs,^[^ ^48^ ^]^ both available online (MATLAB central file exchange). Gated population data were imported in OriginPro (OriginLab) for plotting and further (statistical) analysis.

### Figure Preparation and Statistics

Schematics were created using Adobe Illustrator. Schematic depiction of the confocal microscope and the EVOS microscope were obtained from BioRender.com. All graphs were created using MATLAB software or OriginPro (OriginLab) software. The latter was also used for fitting and statistical analysis. Box plots show the mean (line) and standard deviation (error bars). Boxes indicate the range of the 25–75th percentile. The type of statistical test used is indicated in the figure description. Image analysis, including 3D visualization, was performed with either Fiji (ImageJ) or Imaris (Oxford instruments).

## Conflict of Interest

The authors declare no conflict of interest.

## Supporting information



Supporting Information

## Data Availability

The data that support the findings of this study are available from the corresponding author upon reasonable request.
