# Supplementary material for: High‐Throughput Single‐Cell Analysis of Local Nascent Protein Deposition in 3D Microenvironments via Extracellular Protein Identification Cytometry (EPIC)
Source: Adv Mater. 2024 Dec 4;37(6):2415981. doi: 10.1002/adma.202415981 (PMC11817916; doi:10.1002/adma.202415981)
Supplement: Supplementary file 1 — Supporting Information [file ADMA-37-2415981-s001.pdf]

ADVANCED MATERIALS

Supporting Information

for *Adv. Mater.*, DOI 10.1002/adma.202415981

High-Throughput Single-Cell Analysis of Local Nascent Protein Deposition in 3D Microenvironments via Extracellular Protein Identification Cytometry (EPIC)

*Marieke Meteling, Castro Johnbosco, Alexis Wolfel, Francisco Conceição, Kannan Govindaraj, Liliana Moreira Teixeira and Jeroen Leijten**

Supporting Information

High-Throughput Single-Cell Analysis of Local Nascent Protein Deposition in 3D Microenvironments via Extracellular Protein Identification Cytometry (EPIC)

*Marieke Meteling[1], Castro Johnbosco[1], Alexis Wolfel[1], Francisco Conceição[2], Kannan Govindaraj[1], Liliana Moreira-Teixeira[2], and Jeroen Leijten[1]**

Supplementary Methods

Immunostaining of embedded tissue sections: Small sections of human subchondral bone tissue were fixated in 10% formalin overnight, and subsequently embedded in paraffin according to standard procedures. Embedded tissues were cut into slices of 5 µm thickness, and bonded to a glass slide. Tissue were deparaffinized following standard procedure. For immunostaining, sections were first incubated with a 1:1 mixture of 0.1% w/v hyaluronidase in PBS combined with 5 µg ml^{-1} Proteinase K at 37 °C for 30 min. After washing with PBS, samples were blocked with a 3% BSA solution at RT for 1h. Sections were incubated with primary antibodies for COL I, COL IV, and COL VI, or the respective rabbit isotype IgGs in blocking buffer (3% BSA dissolved in PBS) at RT for 1 h. Same dilutions as for the encapsulated cells were used. Washing was performed thrice using 0.1% PBS-T (washing buffer). Sections were incubated with respective secondary antibody Alexa Fluor 488 (dilution 1:300), including a 2nd AB control sample, in blocking buffer at RT for 30 min. Afterwards, sections were washed again thrice with washing buffer. Last, DAPI staining was added (1:500), and incubated at RT for 5 min. Sections were washed with PBS once, then immersed in tap water. After air drying, sections were mounted with fluorescent mounting medium and cover slips.

Pellet culture, embedding and immunostaining: hPCs were cultured as described above. For pellet culture, the cells were trypsinized, counted, and seeded into either a 96 round bottom well plate or a 15 ml conical tube. Per well/tube 0.2 mil cells were seeded. Cells were centrifugated to create pellets. The pellets were transferred to chondrogenic medium (same composition as for the encapsulated cells) and subsequently incubated at 37°C and 5% CO_2. Medium was refreshed thrice a week. Following three weeks of culture, the pellets were fixated using 4% PFA. Samples were washed and incubated with 0.1% PBS-T for permeabilization. As

preparation for cryoembedding, samples were incubated in 15% sucrose solution until the pellets sedimented. The same was repeated in 30% sucrose solution. Subsequently, samples were embedded in cryomatrix, and then directly frozen by completely immersing them in cold isopentane, which was stored at -80°C. Frozen samples were stored till sectioning at -80°C. Cryosectioning was performed according to standard procedure. Cut sections were transferred to superfrost glass slides. The glass slides were left to dry at RT. For antigen retrieval, a 1:1 mixture of hyaluronidase and proteinase K was used (same concentrations as for the microgels). Incubation was performed under humid conditions at 37°C for 30 min. Afterward, sections were washed twice with 0.1% PBS-T, followed by a PBS wash. They were then blocked with 5% BSA at RT for 30 min. Sections were incubated with primary antibodies (COL I, COL IV, COL VI or FN) at the same dilutions as for the microgels. Incubation in blocking buffer at RT for 1 h. Washing was performed thrice with 0.1% PBS-T, followed by incubation with the secondary antibody (AF488 or AF647; 1:300 dilution) in blocking solution at RT for 1 h. Sections were washed again with 0.1% PBS-T. For nuclear counterstaining, DAPI staining (1:500; 1% BSA) was added to the slides. Incubation at RT for 5 min. Finally, sections were washed once in PBS, then transferred to running tap water, and dried at 37°C for 5 min. Dried sections were mounted with fluorescent mounting medium and cover slips. Samples were stored at 4°C till imaging was performed.

COL I diffusion assays: Spiked COL I microgels were produced as described in the method section of the manuscript, employing a COL I concentration of 0.025 µg µl^{-1}. To study the diffusion of the spiked-in COL I out of the microgels, generated beads were retrieved from the oil phase and subsequently split in two batches. The first batch was directly stained for COL I using immunostaining, while the second batch was incubated for three days prior to

immunostaining. An isotype staining for the COL I antibody served as a control. Stained microgels were imaged using a confocal microscope (Zeiss LSM 880) with a 40x water objective. Additionally, pure Dex-TA microgels were created, which were incubated in COL I protein (1 mg ml^{-1}) solution overnight, to allow COL I to diffuse into the microgels. The microgels were transferred to an excess amount of PBS the next day, and the microgels were divided into two batches. The first batch was fixated with 4% formol, and subsequently immunostained for COL I. The second batch remained in PBS solution, and was fixated and stained after three days to allow for diffusion of the COL I out of the microgels. As a control for the antibody staining, an isotype staining for the COL I antibody was used. Additionally, pristine Dex-TA microgels (e.g., not incubated with COL I protein), were stained for COL I as negative control. Stained microgels were imaged using a fluorescent confocal microscope (Zeiss LSM 880) with a 40x water objective. In both instances, z-stack imaging was performed to image the entire microgel. Z-stacks were analyzed using ImageJ by generating MIP images from the z-stacks.

Immunostaining of non-encapsulated cells: hPCs were cultured in monolayer in growth medium for one week. Subsequently, cells were harvested using 0.25% Trypsin-EDTA solution and fixated in Formol (4%). After washing in PBS, the cells were blocked in 5% BSA solution for 1 h at RT under dynamic conditions. Subsequently, cells were stained for COL I, COL IV, COL VI, or FN, or any of the respective isotype controls, using identical antibody concentrations as with encapsulated cells. Incubation was performed for 2h at RT under dynamic conditions. Washing was performed once using 0.5% PBS-T and twice using 5% BSA. Subsequently, samples were incubated with their respective secondary antibody labelled with Alexa Fluor 488 or Alexa Fluor 647 (both 1:300) in blocking buffer under dynamic conditions

at RT for 2h. Samples were washed as described before. For flow cytometry analysis, cells were resuspended in 1.5% BSA and passed through a 40 µm cell stainer. Additionally, stained cells were also imaged. To this end, antibody-stained cells were additionally stained with DAPI (nuclei stain) and phalloidin Alexa Fluor 488 or phalloidin Alexa Fluor 647 (actin stain), depending on the secondary antibody used. To facilitate imaging, samples were resuspended in PBS and transferred to a µ-slide dish (Ibidi) for high resolution imaging. Z-stacks were obtained using a fluorescent confocal microscope (Zeiss LSM 880) with a 40x objective. MIP image compilation and pseudo-coloring was achieved using Fiji (ImageJ).

Cell embedding in bulk hydrogel and nascent protein staining: To investigate the heterogeneity in matrix deposition inside a bulk hydrogel, primary human chondrocytes were embedded in a hydrogel at a density of 2 million cells ml^{-1}. For nascent protein visualization, cells were cultured for one week in chondrogenic medium using a methionine-free version of DMEM, which was supplemented with a methionine analogue azidohomoalanine (AHA, Jena Bioscience). Subsequently, newly deposited nascent proteins were specifically labelled with a fluorophore-conjugated cyclooctyne (DBCO-488, Jena Bioscience) that covalently binds to AHA in the protein backbone through Click-It chemistry.[1] Samples were washed with 0.1% BSA, and incubated with 30 uM DBCO-488 at 37°C for 30 min. After three washes with 0.1% BSA, cells were fixated with 4% PFA, followed by permeabilization using 0.1% Triton X-100. Samples were blocked with 0.1% BSA. Actin filaments were stained using phalloidin-594 (1:100 dilution, Biolegend) in blocking solution overnight at 4°C. After three washes with 0.1% BSA, nuclei were stained with DAPI (1:1000) at RT for 15 min. Z-stacks from bulk hydrogels were obtained using a fluorescent confocal microscope (Zeiss LSM 880) with a 20x objective.

Image analysis was performed using Fiji (ImageJ). For intensity analysis, MIPs were reconstructed of part of the stack, and only cells within the same focus plane were analyzed.

Supplementary Figures

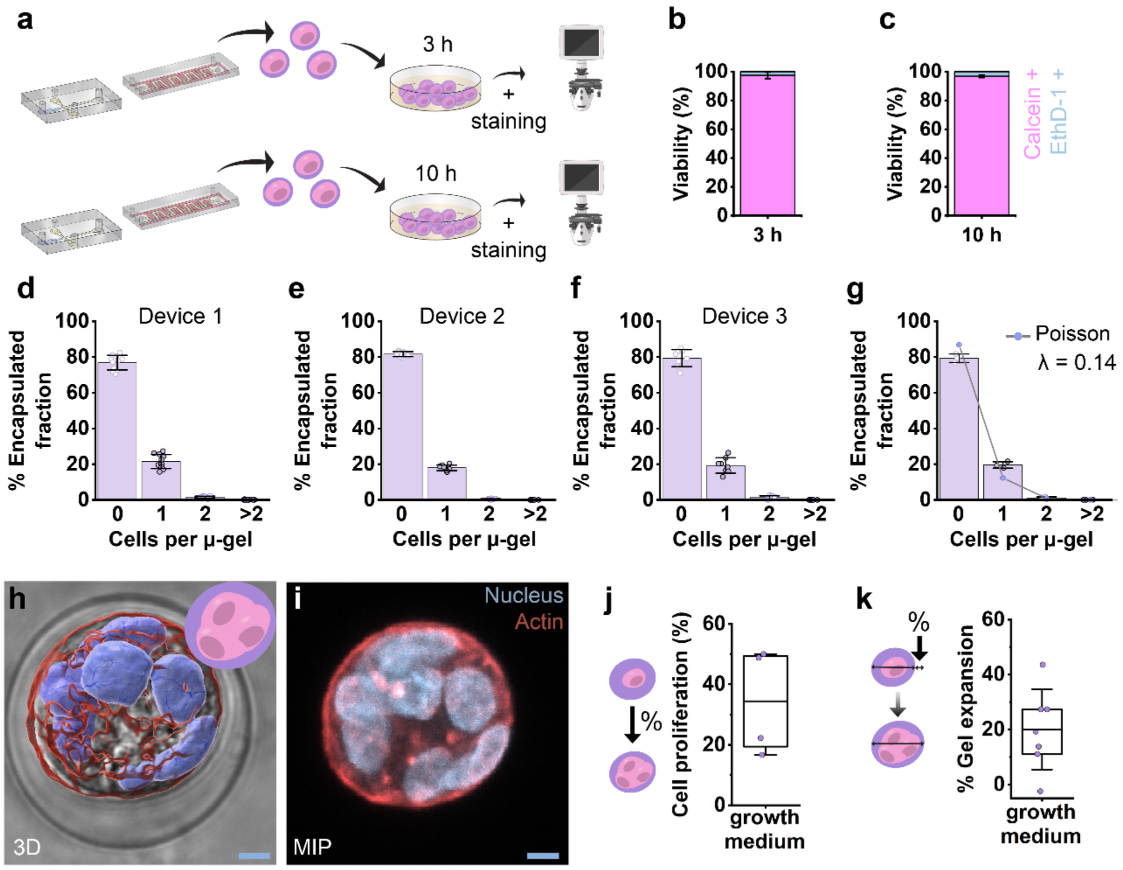

Figure S1: *a) Semi-quantification of cell viability based on Calcein AM (live; pink) and EthD-1 staining (dead; blue) on day 0 at different time points: b) 3 h after encapsulation, and c) 10 h after encapsulation. Encapsulated fraction of cells per microgel quantified across three different devices/independent experiments on day 0. Semi-quantification of encapsulated fractions per device and experiment: d) device 1 (n = 10 images; n = 4942 µ-gels), e) device 2 (n = 8 images; n = 2930 µ-gels), f) device 3 (n= 8 images; n = 1061 µ-gels), g) Mean fraction per device plotted together with the theoretical Poisson values (n = 3 experiments/ sets of chips). Confocal micrographs showing encapsulated hPCs in growth medium, which proliferated inside the microgel: h) Overlay of confocal image (midsection, bright field) and respective 3D reconstruction created with Imaris. i) Confocal fluorescent MIP image of the same aggregate. j) Semi-quantification of the cell proliferation inside microgels for encapsulated cells cultured in growth medium. k) Microgel expansion in response to cell proliferation during culture in growth medium. Statistics: The lines in the box plots and the*

bars indicate the mean, the error bars give the standard deviation. Boxes indicates the 25th and 75th percentile. Scale bars represent 5 µm, MIP – maximum intensity projection.

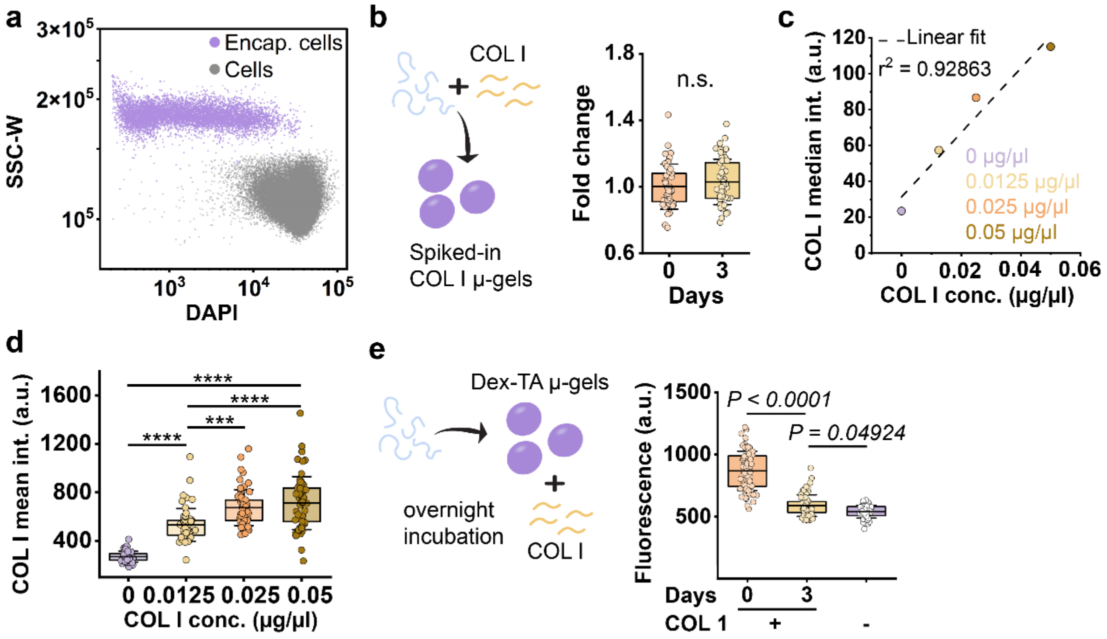

***Figure S2:** a) DAPI signal plotted against side scatter (SSD-W), showing two distinct populations: pristine cells (grey) and cells microencapsulated in Dex-TA microgels (purple) (n = 56171 cells, n = 7527 encap. cells). b) COL I intensity of COL I spiked microgels comparing measurements after 0 and 3 days, normalized to the sample directly stained and measured after microgel production (n = 41 µ-gels (day 0), n = 50 µ-gels (day 3)). c) Linear relation between median signal of spiked COL I microgels and corresponding COL I concentration inside the microgels based on flow cytometry analysis. d) Confocal micrograph analysis of COL I spiked microgels: COL I mean intensity data per microgel plotted for each condition (n = 42 (0 µg/µl), n= 57 (0.0125 µg/µl), n= 63 (0.025 µg/µl), n= 61 (0.05 µg/µl) µ-gels per condition). e) Dex-TA microgels incubated in COL I solution overnight, which were either directly stained or after three days incubation in an excess amount of PBS. Pristine Dex-TA microgels (e.g., not incubated with COL I protein) was used as a negative control (n = 66 µ-gels (day 0), n = 66 µ-gels (day 3), n = 64 µ-gels (neg. control)). Statistics: The lines in the box plots indicate the mean. Error bars give the standard deviation. Boxes indicates the 25th and 75th percentile. Kruskal-Wallis test with Dunn's post hoc test, *** p < 0.001, **** p < 0.0001, n.s. – non significant.*

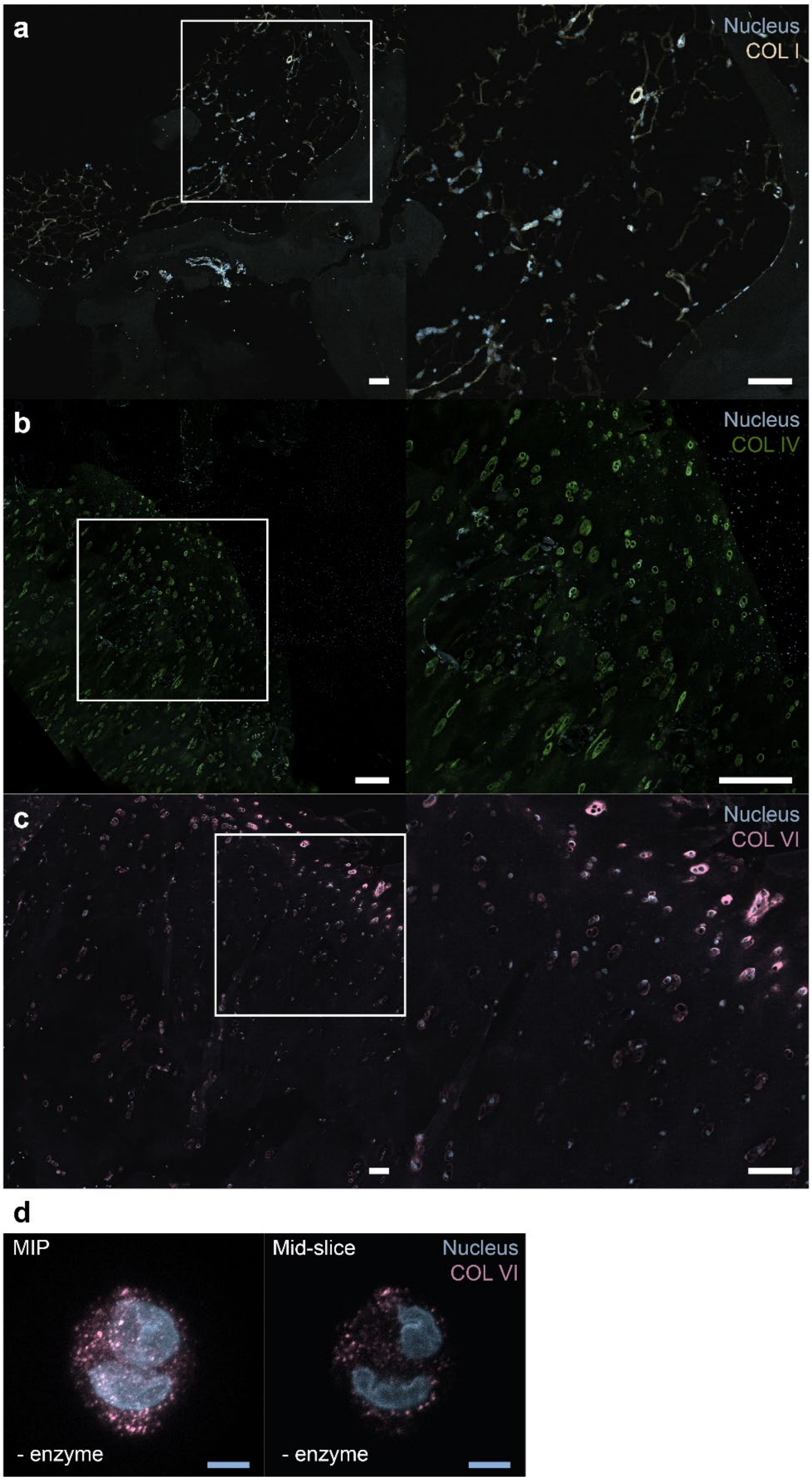

Figure S3: *Confocal fluorescent tile scans (midsection) of human subchondral bone sections stained for a) COL I, b) COL IV, c) COL VI. Nuclear counterstaining was performed with DAPI (blue). White rectangle on the left indicates the zoomed-in section on the right side. d) Confocal*

MIP and mid-slice image of COL VI stained encapsulated cell without enzymatic epitope retrieval treatment. Scale bars: white – 200 µm, blue – 5 µm.

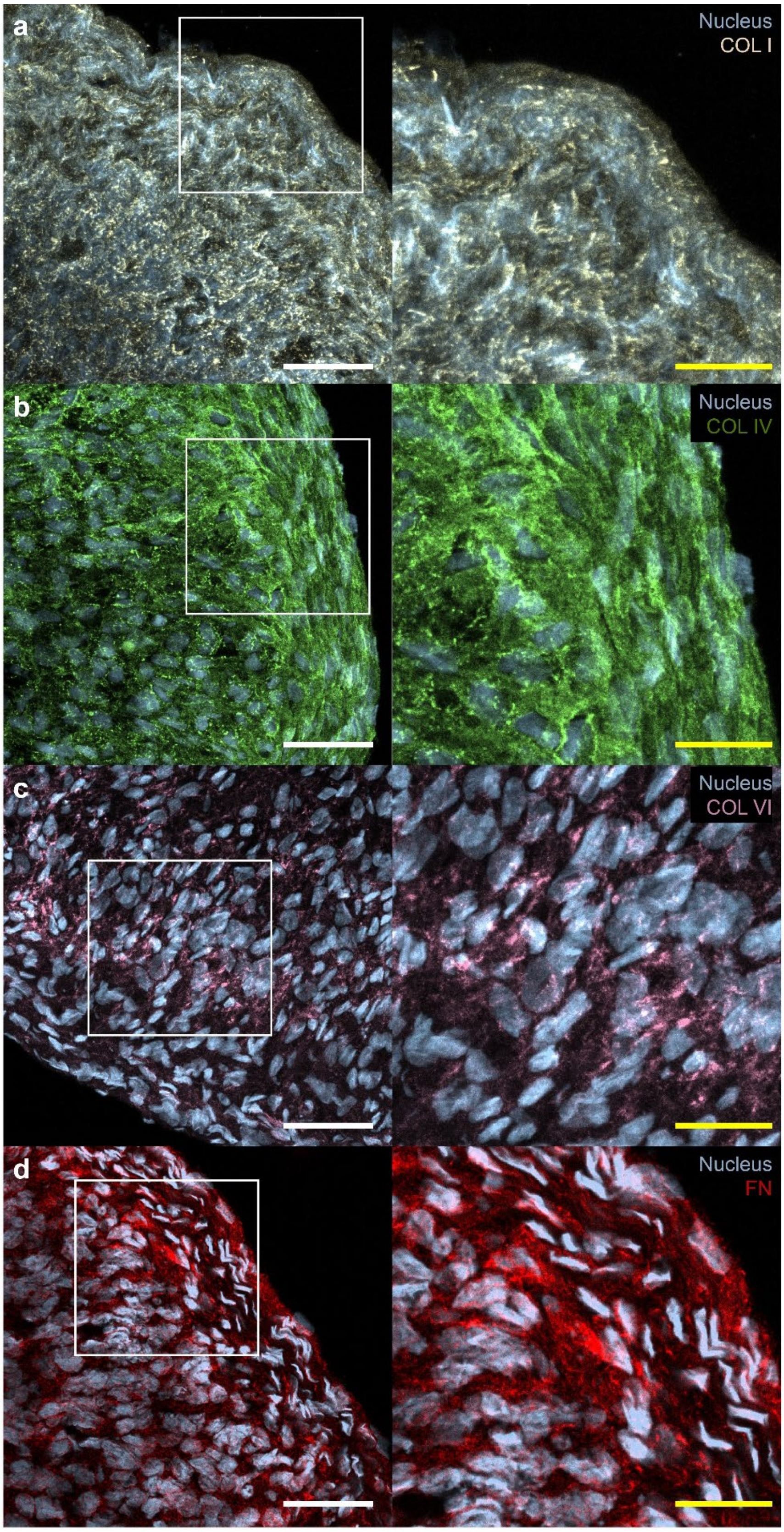

Figure S4: *Confocal fluorescent micrographs of cryosectioned hPC pellets culture in chondrogenic medium for three weeks. Showing nuclear staining (blue) and immunostaining for a) COL I (yellow), b) COL IV (green), c) COL VI (pink), d) FN (red). White rectangle shows the area of the zoomed-in region shown on the right side. Scale bars: white – 200 µm, yellow – 100 µm*

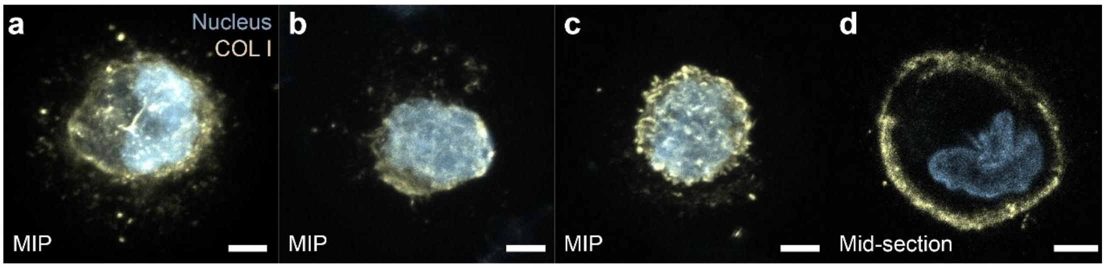

Figure S5: *Confocal fluorescent micrographs of encapsulated hPCs cultured for three weeks, stained for nucleus (blue) and immunolabelled for COL I (yellow): a-c) Maximum intensity projection images, d) Mid-section of an encapsulated cell. Images were enhanced in ImageJ to show the penetration of COL I into the microgel. Scale bars represent 5 µm*

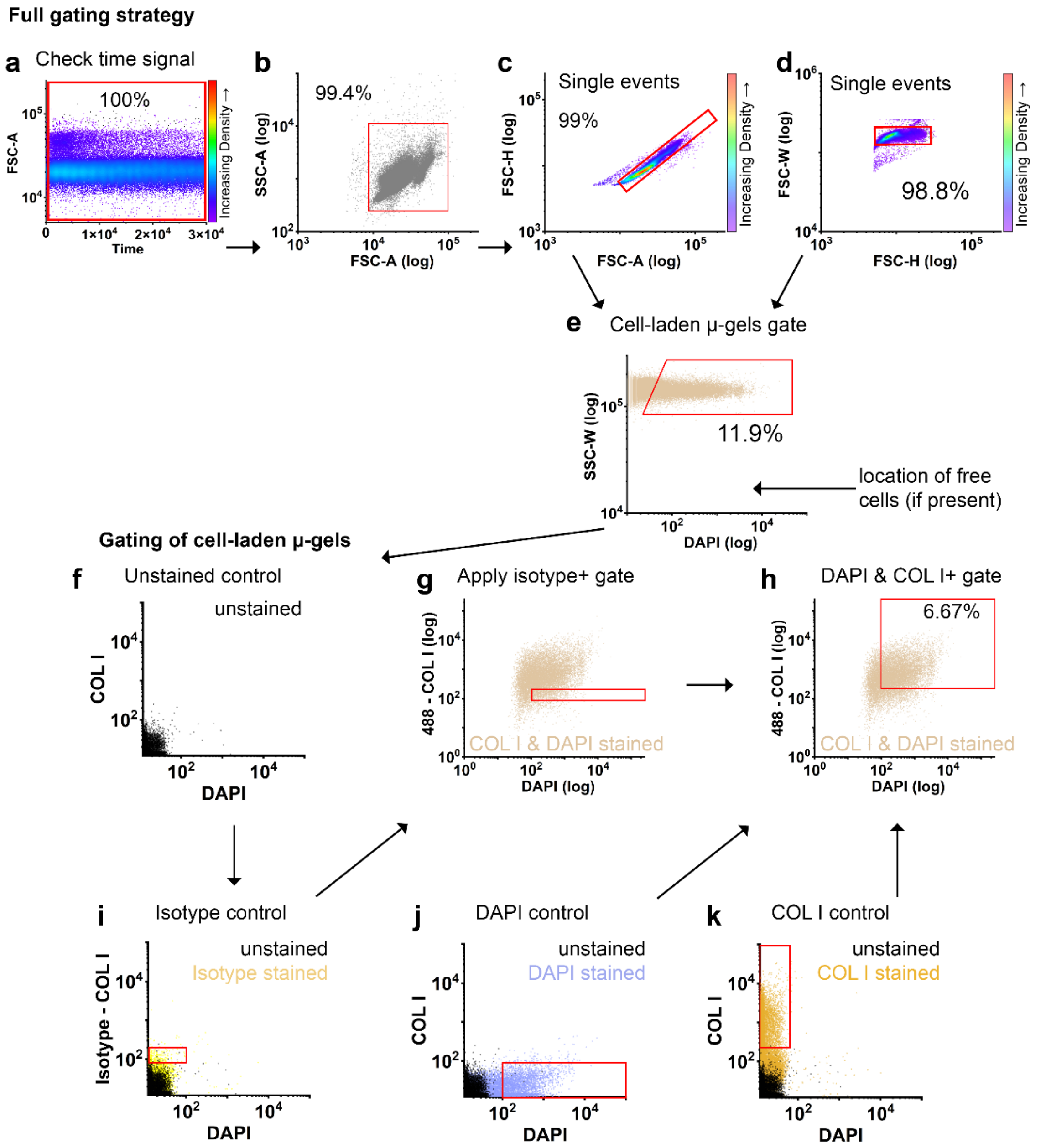

Figure S6: *Example showing gating strategy for EPIC: a) Data is checked for sample uptake (time signal), b) FSC-A and SSC-A gate is employed to exclude debris, c)-d) gating is used to identify single events. e) Gating is designed to identify cell-laden microgels based on DAPI signal and SSC-W signal. Gating of the identified cell-laden microgels: f) Unstained cell-laden microgels are used to determine the "negative" signal. g) DAPI signal is plotted against the matrix signal (here COL I) of COL I and DAPI stained encapsulated hPCs. Gate for the isotype and DAPI positive cell-laden microgel population is shown (red rectangle; this population is considered COL I- DAPI+). h) COL I and DAPI signal of COL I and DAPI stained hPCs, with inside the gate (red rectangle) the COL I+ DAPI+ population. Controls: i) Isotype control (for*

COL I) stained encapsulated hPCs. The signal is used to determine the "isotype gate" (shown in g). j) DAPI stained encapsulated hPCs with respective DAPI gate. k) COL I stained encapsulated hPCs with respective COL I gate.

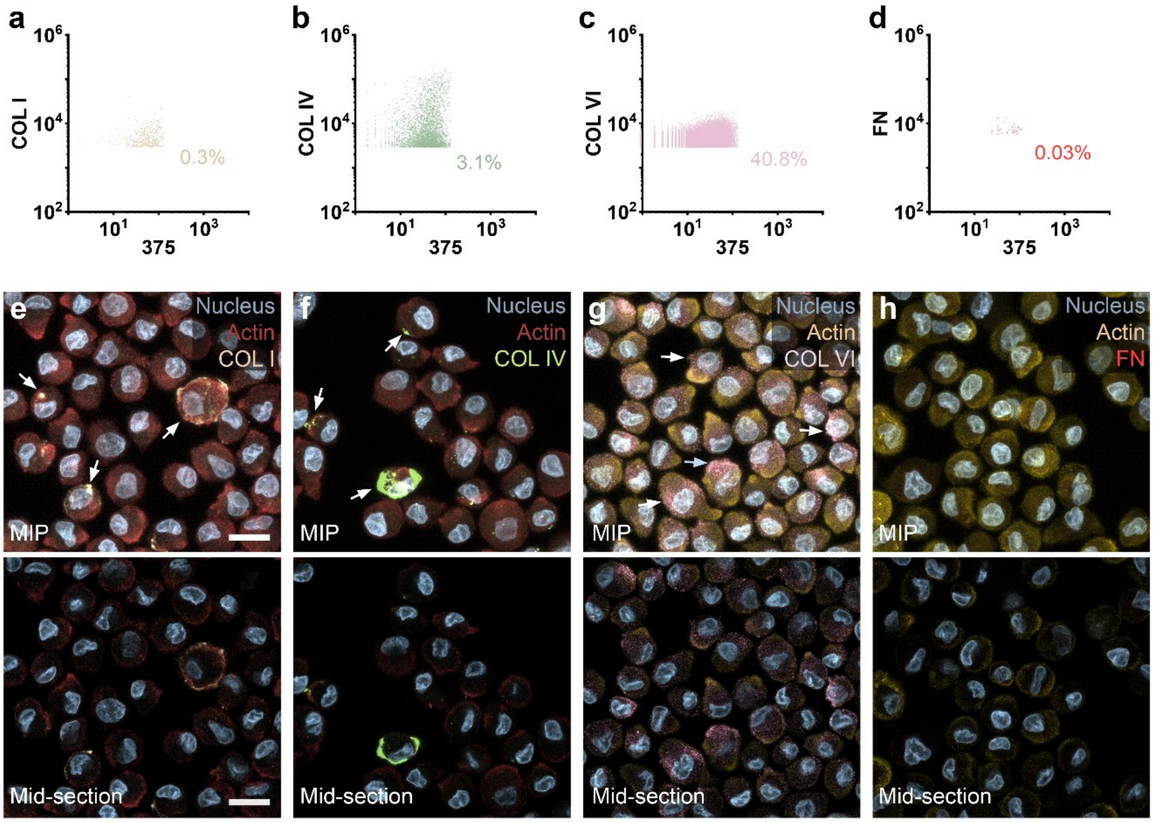

Figure S7: *Trypsinized hPCs (non-encapsulated) stained for ECM proteins to test for incomplete removal of the respective ECM proteins after trypsinization. Flow cytometry graphs showing the positive population of a) COL I (yellow), b) COL IV (green), c) COL VI (pink), and d) FN (red). Percentage of positive population is stated in-graph. Corresponding confocal micrographs of hPCs, additionally stained for the nuclei (blue) and the actin cytoskeleton (red or orange), showing e) COL I (yellow) stained hPCs, f) COL IV (green) stained hPCs, g) COL VI (pink) stained hPCs, h) FN (red) stained hPCs. Upper panels show confocal z-stack based MIP, the lower panels the corresponding mid-section through the cells, revealing intracellular COL VI staining. Arrows (white) indicate remaining ECM proteins on the cells (if any), or intracellular ECM proteins. Scale bars represent 20 µm.*

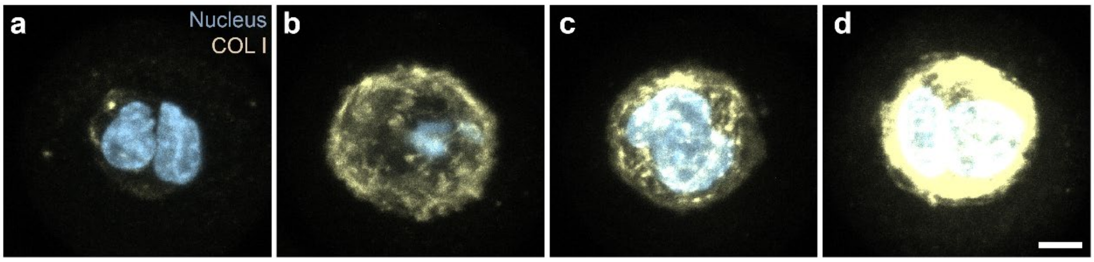

Figure S8: *a)-d) Confocal fluorescent micrographs (MIPs) of encapsulated hPCs cultured for three weeks in chondrogenic medium, all originating from the same batch. Stained for COL I (yellow) and the nucleus (blue), showing variation in COL I deposition. Images were not digitally enhanced, and were all taken using identical confocal microscopy settings. Scale bar represents 5 µm, MIP – maximum intensity projection.*

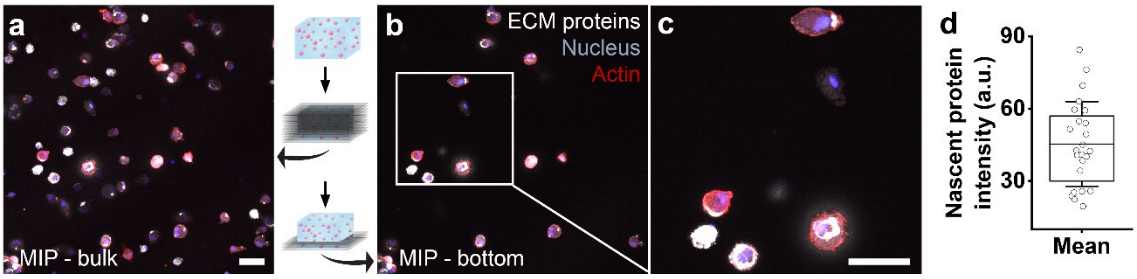

Figure S9: *Chondrocytes embedded in bulk hydrogel and cultured for one week. Metabolic labeling of nascent matrix proteins was performed. a) Confocal z-stack based MIP of entire hydrogel showing the nuclei (blue), the actin cytoskeleton (red), and the deposited nascent proteins (white). b) Confocal z-stack based MIP of the bottom part of the bulk hydrogel closest to the objective. The stack size was chosen to include one layer of cells. c) Magnification of the area indicated with the white square in image b, showing large variations in nascent protein deposition (white). d) Semi-quantification of nascent protein deposition based on the MIP images of the bottom of the bulk hydrogel. Only cells within the plane of focus were taken along for quantification. Statistics: Error bars give standard deviation, line in the box plot gives mean. Boxes represent the 25th and 75th percentile. Scale bar represents 20 µm.*

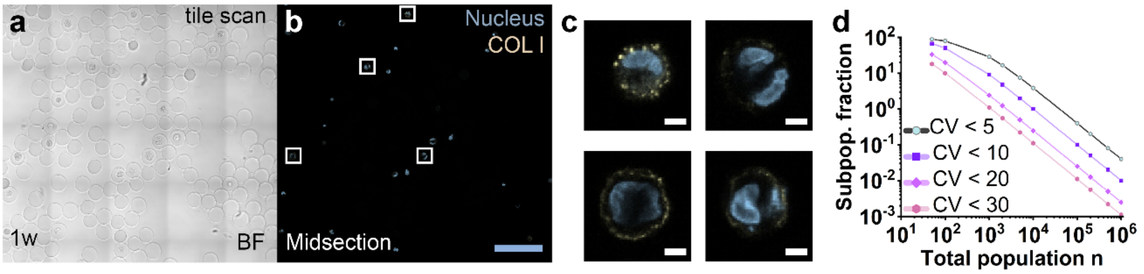

Figure S10: *a-c) Tile scan of microgels cultured for one week and subsequently stained for COL I (yellow) and the nucleus (blue): a) Confocal bright field image (midsection, tile scan) and respective b) fluorescent confocal micrograph are shown, with c) selected digitally zoomed-in regions (indicated with white squares in b) showing single microgels and variation in COL I deposition of encapsulated hPCs following one week of culture. d) Relation between the smallest subpopulation fraction to be identified and the total population analyzed regarding different coefficients of variation (CVs). Scale bars: white – 5 µm, blue – 100 µm.*

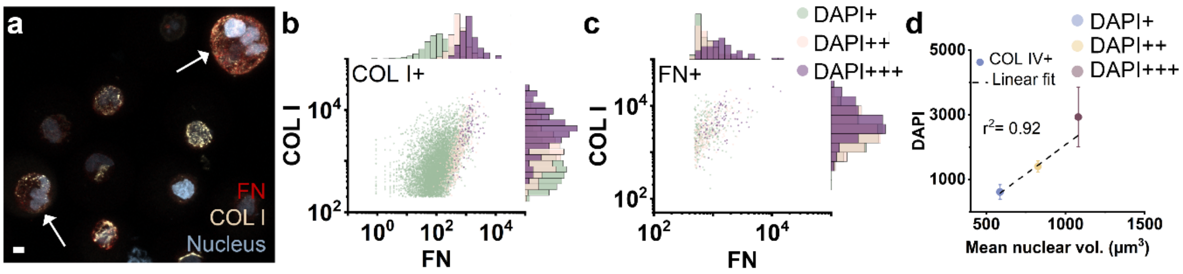

Figure S11: *a) Confocal fluorescent micrograph (MIP) of encapsulated hPCs stained for COL I (yellow), FN (red) and the nucleus (blue). The microgels were sorted employing FACS (COL I+ DAPI+). Within the sorted microgel population microgels with more than one cell inside are present (arrows). b)-c) Flow cytometry data of COL I and FN stained encapsulated hPCs. Scatter plots with histogram indicating the distribution of the three different DAPI subpopulations (DAPI+, DAPI++, DAPI+++), shown for the b) DAPI+ COLI+ population, and the c) DAPI+ FN+ population. d) Average nuclear volume of single-cell hPC microgels, stained for COL IV and FN, for the three different DAPI subpopulations, plotted against the corresponding average DAPI signal (for the COL IV+ population) obtained by flow cytometry analysis. Linear fitting was performed, giving an adjusted r^2 = 0.92. Error bars show the standard deviation. Scale bar: 5 µm. MIP – maximum intensity projection.*

References

[1] C. Loebel, R. L. Mauck, J. A. Burdick, *Nat Mater* **2019**.
